# *Drosophila suzukii*: The Genetic Footprint of a Recent, Worldwide Invasion

**DOI:** 10.1093/molbev/msu246

**Published:** 2014-08-25

**Authors:** Jeffrey R. Adrion, Athanasios Kousathanas, Marta Pascual, Hannah J. Burrack, Nick M. Haddad, Alan O. Bergland, Heather Machado, Timothy B. Sackton, Todd A. Schlenke, Masayoshi Watada, Daniel Wegmann, Nadia D. Singh

**Affiliations:** ^1^Department of Biology, Indiana University, Bloomington; ^2^Department of Biology, University of Fribourg, Fribourg, Switzerland; ^3^Swiss Institute of Bioinformatics, Lausanne, Switzerland; ^4^Department of Genetics and IRBio, University of Barcelona, Barcelona, Spain; ^5^Department of Entomology, North Carolina State University; ^6^Department of Biological Sciences, North Carolina State University; ^7^Department of Biology, Stanford University; ^8^Department of Organismic and Evolutionary Biology, Harvard University; ^9^Department of Biology, Reed College, Portland, OR; ^10^Department of Biology, Ehime University, Matsuyama, Ehime, Japan

**Keywords:** *Drosophila suzukii*, population genetics, invasion, pest

## Abstract

Native to Asia, the soft-skinned fruit pest *Drosophila suzukii* has recently invaded the United States and Europe. The eastern United States represents the most recent expansion of their range, and presents an opportunity to test alternative models of colonization history. Here, we investigate the genetic population structure of this invasive fruit fly, with a focus on the eastern United States. We sequenced six X-linked gene fragments from 246 individuals collected from a total of 12 populations. We examine patterns of genetic diversity within and between populations and explore alternative colonization scenarios using approximate Bayesian computation. Our results indicate high levels of nucleotide diversity in this species and suggest that the recent invasions of Europe and the continental United States are independent demographic events. More broadly speaking, our results highlight the importance of integrating population structure into demographic models, particularly when attempting to reconstruct invasion histories. Finally, our simulation results illustrate the general challenge in reconstructing invasion histories using genetic data and suggest that genome-level data are often required to distinguish among alternative demographic scenarios.

## Introduction

Recent history is rife with examples of biological invasions of both terrestrial and aquatic species. Many of these invasions are thought to be human mediated, precipitated in part by dramatic increases in global trade (Westphal et al. 2008). These invasions can pose serious threats to biodiversity; at least 98% of imperiled Hawaiian birds and plants, for instance, are threatened at least in part by invasive species. Although island ecosystems may be particularly at risk for the deleterious effects of invasive species, the threats posed by invasive species to native ecosystems are certainly not unique to islands. In the continental United States, 48% of imperiled bird species and 30% of imperiled plant species are threatened at least in part by invasive species (Wilcove et al. 1998).

*Drosophila suzukii* is emerging as a global threat due to both its recent range expansion and the economic impact of colonized populations. *D. suzukii* is presumed to be native to Asia; it was first reported in Japan (Kanzawa 1939) and has been recorded in several other parts of Asia as well (Calabria et al. 2012; Cini et al. 2012). In the 1980s, *D. suzukii* successfully invaded Hawaii (Kaneshiro 1983; O'Grady et al. 2002). In 2008, this species was reported in both California and Spain (Hauser 2011; Calabria et al. 2012) and has since expanded throughout North America and Europe (Hauser 2011; Burrack et al. 2012; Cini et al. 2012). Unlike other *Drosophila* species, *D. suzukii* is an economically damaging pest because females clearly prefer ripening fruit to rotting fruit (Mitsui et al. 2006) and oviposition preference appears to be associated with the onset of fruit coloration for a wide variety of host plants (Lee et al. 2011). Estimates of the economic burden associated with this pest due to crop loss and control efforts are dramatic in both Europe and North America (Bolda et al. 2010; Goodhue et al. 2011; Cini et al. 2012), highlighting the importance of managing this newly invasive pest.

In spite of the threat posed by *D. suzukii*, almost nothing is known regarding its colonization history. Population genetics offers much promise for both understanding and managing new and high impact invasive species in general and for *D. suzukii* in particular (for review see Estoup and Guillemaud 2010; also see Fitzpatrick et al. 2012). For instance, population genetic approaches have been used to reconstruct colonization routes in a number of invasive species (Nardi et al. 2005; Pascual et al. 2007; Rius et al. 2012). Identifying source populations and major colonization routes can empower management strategies, as these represent targets for control efforts. Identifying source populations is also important in that understanding the natural history of *D. suzukii* in its native environment might also suggest new control strategies, such as the types of natural parasites that might serve as effective biocontrol agents (Roderick and Navajas 2003). Population genetic approaches have the potential to discriminate between single and recurrent introductions; while management efforts in the former scenario might focus on eradication, recurrent introductions require a different strategy. In the case of *D. suzukii*, the source(s) of the continental US and European invasions are unknown. Similarly, it has yet to be determined whether these recent invasions represent independent colonization events. Moreover, it is unclear how populations within the continental United States are genetically related. Analysis of population structure in this area may indicate pathways of spread, and thus identify targets for reducing spread.

More broadly speaking, a species’ ability to compete with endemic species (Pascual et al. 1998, 2000) and to adapt to new environments likely plays a significant role in its probability of success as an invader (Balanya et al. 2006). Adaptation requires genetic variation and as a consequence, insight into levels of genetic diversity in native versus derived populations is critical for understanding the success of biological invasions. Although preliminary data are suggestive of high levels of genetic diversity in a California sample of *D. suzukii* (Carvajal 2010), the extent of founder effects in the US and European invasions remains unknown.

Here, we use a population genetic approach to 1) characterize patterns of genetic diversity in *D. suzukii* and 2) shed light on the colonization history of this emergent invasive species with a particular focus on the US invasion. Our worldwide sample includes one population from Japan (in the presumed ancestral range), one population from Hawaii (early invasion), nine continental US samples (recent invasion), and one European sample (recent invasion). Our multilocus survey of patterns of genetic diversity in this species reveals high levels of genetic diversity in colonizing populations. This high level of diversity may contribute to the success and expansion of these populations. Our data are also suggestive that Europe and the continental United States were invaded independently.

Of more general importance, our use of approximate Bayesian computation (ABC) to reconstruct the demographic history of this species reveals that integrating population structure into models is of paramount importance for demographic inference, particularly in the case of invasive species. In addition, our results suggest that the power to distinguish among alternative demographic scenarios for invasive species is limited and that resolving complex demographic histories requires genome-wide surveys of genetic variation.

## Results and Discussion

### Genetic Diversity

**Fig. 1.**
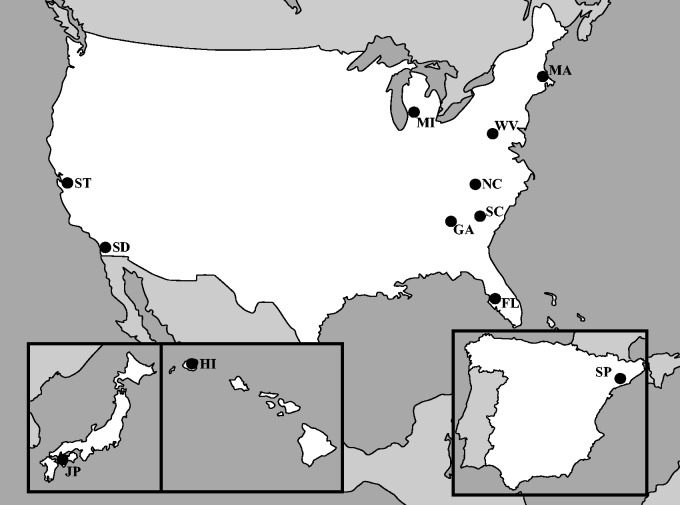
Map depicting the 12 sampling locations. Each sample is noted by its two letter code (see Materials and Methods).

We surveyed genetic diversity in 246 individuals from a global sample of 12 populations (fig. 1) of *D. suzukii* at 6 putatively X-linked loci. Sample sizes ranged from 7 to 24 per population per locus owing to variability in the number of males collected per site as well as variable polymerase chain reaction (PCR) efficacy within loci among individuals. We saw no evidence of heterozygosity within individual samples, which is consistent with X-linkage of these loci. After concatenating the six loci into a single sequence, we calculated nucleotide diversity (*π*, the average pairwise distance among alleles) for all sites and noncoding sites separately (table 1). We potentially have more power to estimate *π* by using all sites than by using only the noncoding sites. However, *π* for noncoding sites, which may evolve more neutrally than sites in coding regions, may be a better indicator of neutral diversity.

**Table 1.** Nucleotide Diversity (*π*) for Each Population Calculated for All Sites and Noncoding Sites Only.

Area	Population	All Sites		Noncoding Sites	
		% *π* [SE]	% Change *π*	% *π* [SE]	% Change *π*
Japan	JP	1.68 [0.140]	—	2.42 [0.228]	—
Hawaii	HI	1.35 [0.147]	−19.64**	1.83 [0.240]	−24.33*
Western United States	ST	1.69 [0.158]	0.36	2.43 [0.253]	0.62
	SD	1.69 [0.160]	0.47	2.53 [0.264]	4.55
Eastern United States	FL	2.28 [0.259]	35.88**	3.49 [0.410]	44.45**
	GA	1.62 [0.148]	−3.55	2.36 [0.244]	−2.26
	MA	1.42 [0.218]	−15.40**	3.11 [0.541]	28.47**
	MI	1.71 [0.154]	1.66	2.49 [0.241]	3.07
	NC	1.51 [0.138]	−10.29**	1.98 [0.212]	−17.93*
	SC	1.82 [0.174]	8.47	2.70 [0.264]	11.51
	WV	1.59 [0.151]	−5.27	2.35 [0.253]	−2.98
Europe	SP	0.88 [0.117]	−47.36**	1.23 [0.177]	−49.00**

*P < 0.05, **P < 0.01 (asterisks denote significance; see Materials and Methods). Standard error is shown in brackets.

Our results provide the first multilocus examination of levels of nucleotide diversity in the emergent invasive species *D. suzukii*. Consistent with previous analysis of mitochondrial gene cytochrome oxidase I (Carvajal 2010; Calabria et al. 2012), the levels of nucleotide diversity in this species are in general quite high. For ease of comparison with other species (in which noncoding diversity is often reported), we focus here on noncoding diversity. At noncoding sites, the levels of nucleotide diversity range from 1.23% (Spain, SP) to 3.49% (Florida, FL) (table 1). Noncoding diversity in JP, a population in the presumed ancestral range of this species, is 2.42%. For comparison, the levels of noncoding diversity at X-linked loci in *D. melanogaster* are approximately 1.2% in Africa (the ancestral range) and 0.48% in Europe (a derived population) (Hutter et al. 2007). An African population of *D. simulans* (likely native to Madagascar; Dean and Ballard 2004) shows the levels of X-linked intron diversity (1.22%) similar to that of *D. melanogaster* (Haddrill et al. 2008). *D. subobscura* provides another useful reference for comparison, as this species recently colonized the Americas from a likely Mediterranean source population (Arauz et al. 2011). Noncoding diversity in this species has been characterized at a few nuclear genes from Mediterranean (SP and Tunisia) populations and estimates range from 0.89% (*Obp83*; Sanchez-Gracia and Rozas 2011) to 1.4% (*rp49* and *Acp70a*, estimated as a weighted average of 5′ and 3′ flanking and intronic sequence; Cirera and Aguade 1998; Rozas et al. 1999), with other genes showing levels of diversity between these two estimates. Thus, at least compared with other species of *Drosophila*, levels of nucleotide diversity in *D. suzukii* appear high.

Biological invasions are often associated with reductions in genetic diversity in colonizing populations (for review see Dlugosch and Parker 2008). However, this is not always the case, as colonizers might retain divergent alleles from their source population. Moreover, recurrent introductions and introductions from different geographic locations can buffer the reduction in genetic diversity (Kolbe et al. 2004; Rius et al. 2012). Comparisons of levels of nucleotide diversity (as measured by *π*) between JP, a population within the presumed ancestral range, and all other (recently derived) populations revealed both increases and decreases in genetic diversity. Overall, levels of nucleotide diversity were not significantly different in derived populations relative to JP in 6 of the 11 derived populations (table 1). This is similar to previous reports in *D. subobscura*; in derived populations, levels of nucleotide diversity are 1.03–1.18%, only slightly smaller than in their ancestral range (1.26–1.30%; Pascual et al. 2007; Arauz et al. 2011) despite the strong bottleneck during the colonization process (Pascual et al. 2007). The five populations that do show significant differences in nucleotide diversity relative to JP are HI and SP, which are discussed in further detail below, and FL, Massachusetts (MA), and North Carolina (NC). Although MA and NC show reduced diversity relative to JP, FL shows higher nucleotide diversity than the Japanese sample (table 1).

**Fig. 2.**
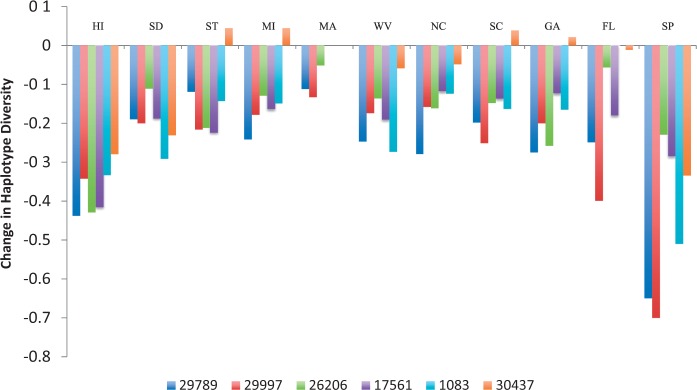
Change in haplotype diversity at each locus in each population relative to Japan as estimated by the following equation:
$$\frac{Hd_{\text{sample}}-Hd_{\text{Japan}}}{Hd_{\text{Japan}}}.$$
.

Although levels of nucleotide diversity show somewhat variable patterns among populations, haplotype diversity shows clear, consistent, and dramatic patterns (fig. 2). Five of the six loci show reductions in haplotype diversity relative to JP in all derived populations. The sole exception is locus 30437, which shows a mix of reductions and increases in haplotype diversity (fig. 2). Overall, haplotype diversities are significantly reduced in derived populations relative to haplotype diversity levels in JP (*P* < 0.03, all populations, Wilcoxon signed-rank test) with the exception of MA (*P* = 0.13, Wilcoxon signed-rank test). Moreover, derived populations show few unique haplotypes, unlike the Japanese population from the ancestral area. This similarity in levels of nucleotide diversity in ancestral versus derived populations contrasted with marked decreases in haplotype diversity and/or unique haplotypes echoes previous findings in other invasive species (Rius et al. 2008). These data may suggest that multiple haplotypes capturing an abundance of nucleotide diversity were carried to the derived populations, but that genetic exchange among haplotypes was limited; this is evidenced by a lack of recombinant haplotype isoforms in the derived populations compared with the presumably ancestral population.

Both the Spanish and Hawaiian populations merit special attention, given that these populations show more pronounced reductions in haplotype diversity than all other populations (fig. 2). Averaged across loci (weighted by locus size), SP and HI show a decrease in haplotype diversity of 43% and 38%, respectively, compared with 10–20% reductions in the remaining populations. This is echoed in patterns of nucleotide diversity, with significant reductions in levels of nucleotide diversity in these populations of 47% and 20% in SP and HI, respectively (table 1). Thus, patterns of genetic diversity in SP and HI are distinct from such patterns in the continental United States, and these two populations appear to be characterized by a marked reduction in genetic diversity relative to JP. These data are consistent with a strong bottleneck in these populations, and are suggestive of a stronger bottleneck in SP than in HI. If the less pronounced bottleneck in HI is due in part to population recovery post-invasion, then this would be consistent with demographic records, as the HI invasion occurred at least 25 years prior to the European invasion (Hauser 2011).

### Population Differentiation

Overall, relative levels of nucleotide diversity at the loci surveyed here are consistent with Japan being a population in the ancestral range, a mild bottleneck in the continental United States and more pronounced bottlenecks in Spain and Hawaii. To examine this further, we examined population differentiation. We first computed pairwise Fst matrices; these results are presented in table 2. As might be expected from patterns of genetic diversity, both HI and SP show strong and significant genetic differentiation from all other populations. This differentiation is consistent with a strong bottleneck effect in these populations, although it should be noted that natural selection and local adaptation at the loci surveyed here could also lead to significant genetic differentiation among populations. There is moderate differentiation between JP and samples both on the East and West Coasts of the United States. However, there is almost no differentiation within the continental United States. The exception is the Stanford sample (ST), which shows significant differentiation from all other continental US samples except San Diego (SD), FL, Georgia (GA), and MA. Given how recent the invasion of the continental United States was (Hauser 2011), it is perhaps unsurprising that there is little differentiation within these continental US samples. However, it is certainly possible that the lack of differentiation is due in part to ongoing migration among populations and/or recurrent invasion.

**Table 2.** Fst Based on Sites for Which at Least Two Individuals Were Sampled Per Population.

Population	JP	HI	ST	SD	FL	GA	MA	MI	NC	SC	WV
HI	0.23*	—									
ST	0.112*	0.042	—								
SD	0.113*	0.118*	0.011	—							
FL	0.032	0.199*	−0.097	−0.352	—						
GA	0.076*	0.195*	0.058	0.037	−0.025	—					
MA	0.027	0.177*	0.05	−0.012	−0.151	0.017	—				
MI	0.036	0.177*	0.075*	0.047	−0.129	−0.028	−0.019	—			
NC	0.061*	0.265*	0.13*	0.061	0.008	0.02	0.042	−0.022	—		
SC	0.111*	0.219*	0.14*	0.02	−0.313	0.061	−0.073	0.052	0.097*	—	
WV	0.076*	0.242*	0.128*	0.021	−0.153	0.024	0.001	0.024	0.006	0.032	—
SP	0.287*	0.491*	0.379*	0.472*	0.535*	0.319*	0.42*	0.285*	0.395*	0.383*	0.363*

Note.—Asterisks denote values significant at P < 0.05 (permutation test, see Materials and Methods).

We conducted two additional tests on the seven populations in the Eastern United States (EUS): MA, West Virginia (WV), NC, South Carolina (SC), GA, FL, and Michigan (MI). First, we tested for any evidence of isolation by distance using a Mantel test. We found no significant positive correlation between genetic (measured here as Fst) and geographic distances (*P* = 0.90, Mantel test), indicating that these populations are not well-described by an isolation-by-distance model. Transforming Fst (see Materials and Methods) did not change these results. The lack of evidence in support of isolation by distance may suggest that the colonization of this area has been mediated passively (e.g., human-mediated transport or perhaps wind) rather than actively through the dispersal capabilities of this species. It may also be that the limited time since colonization and/or a large migration rate contributes to this lack of support for an isolation-by-distance model. We also tested for population differentiation among these seven populations using an analysis of molecular variance (AMOVA) as well as with *ADMIXTURE*. These analyses revealed no significance among population variation component (*P* = 0.51, AMOVA, and supplementary fig. S1, Supplementary Material online), which supports the lack of genetic differentiation among these populations.

### Invasion History

#### Haplotype Relatedness

**Fig. 3.**
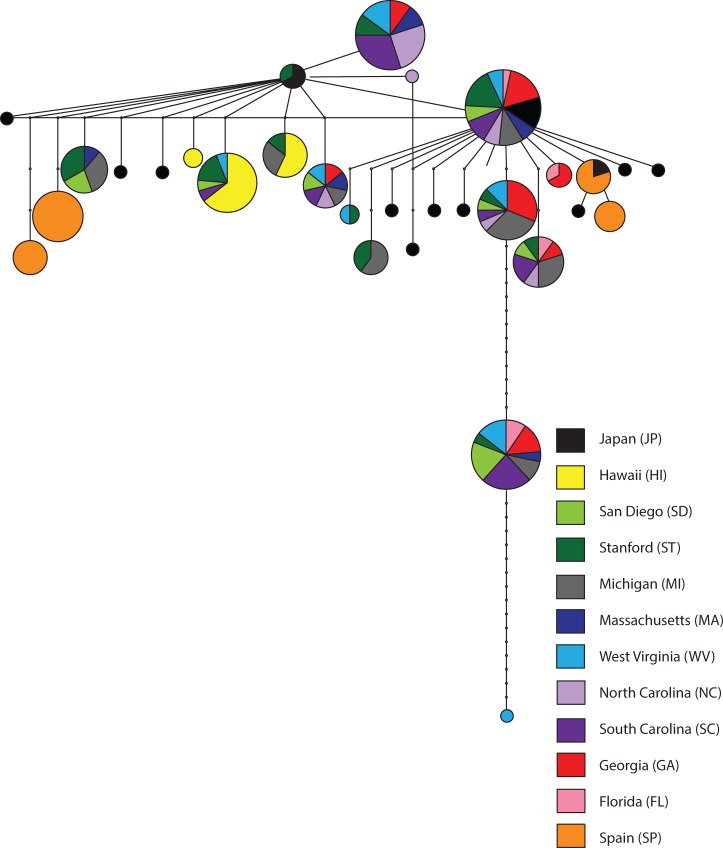
Haplotype network for locus 26206. Each node represents a haplotype and the size of the node is proportional to the number of individuals carrying that haplotype. Edges connecting nodes/vertices denote single mutational steps. Within each node, the individuals carrying each haplotype are shaded by population of origin.

To gain insight into the history of the invasion of the continental United States and Europe, we first examined haplotype relatedness and haplotype sharing among populations considering each locus independently. This analysis was not possible for locus 1083, given the high divergence of the sampled haplotypes. As an example, the haplotype network for locus 26206 is presented in figure 3. Four broad patterns thus emerge from this haplotype network. First, JP has the largest number of unique haplotypes, which is consistent with JP being in the ancestral range of this species. Second, SP and HI have few haplotypes with limited haplotype sharing across the remaining populations. These observations are consistent with both strong founder events in these populations and limited gene flow with the continental United States. Third, the haplotypes found in the continental United States are broadly distributed across these continental US populations indicating a common source for this invasion and/or extensive admixture among these populations. Fourth, the majority of continental US haplotypes are not found outside of the continental United States. This is suggestive that this invasion originated from one or multiple unsampled population(s). It is possible that the US haplotypes arose from new mutations postcolonization, although this is highly unlikely given the recency of the invasion. If another population in the presumed ancestral range of this species served as the source for the European and continental US invasions, this would indicate significant population structure of *D. suzukii* in its native range.

These four broad patterns are recapitulated in the haplotype networks of the other four loci for which this analysis was possible (supplementary figs. S2–S5, Supplementary Material online). Summaries of total haplotype numbers, unique haplotypes (haplotypes present in a single individual), private haplotypes (haplotypes found only in a single population), and shared haplotypes (haplotypes shared among populations) are presented in supplementary figure S6, Supplementary Material online, and highlight the high number of unique haplotypes in JP and the preponderance of private haplotypes in SP and HI.

#### Model Formulation

**Fig. 4.**
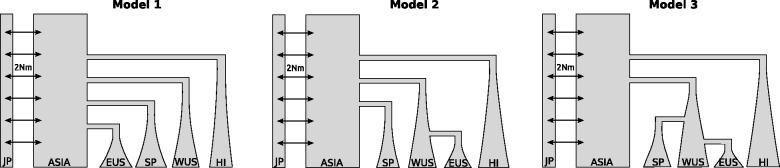
Invasion models for *Drosophila suzukii*. We denote as ASIA the unsampled source population of the invasions and JP, HI, WUS, EUS, SP the Japanese, Hawaii, Western United States, Eastern United States, and Spanish populations for which we have samples. For Model 1, we assumed independent colonization of all continents from Japan. For Model 2, we assumed that EUS was colonized from WUS. For Model 3, we assumed that both the SP and EUS populations were colonized from WUS. The arrows between ASIA and JP denote migration between those populations at a rate equal to 2 Nm.

We next leveraged historical records in combination with the diversity patterns in our data to enumerate possible colonization scenarios of how *D. suzukii* spread from its native range in Asia (termed ASIA in our models) to the four distinct geographical areas: HI, Western United States (WUS; SD and ST), Eastern United States (EUS) (all remaining continental US samples), and SP. Because our haplotype sharing results indicate that HI is an unlikely source for the continental US and European invasions, we treat the colonization of HI as independent from the other populations (fig. 4). For Model 1, we assumed independent colonizations of all regions. For Model 2, we assumed independent colonizations for all regions except for the EUS, which was assumed to be colonized from the WUS. For Model 3, we assumed independent colonizations of the WUS and that the EUS and SP were sequentially colonized from the WUS. This last model was informed by the timing of reports of *D. suzukii* incidence on the west coast of the United States and Europe (Hauser 2011; Calabria et al. 2012). In all three models, we assumed that the invading populations experienced exponential growth after being founded.

Modeling the source population (ASIA) requires particular attention, as only a model that accurately reproduces the genetic diversity in the source population can yield information about the colonization scenarios. We explored five demographic scenarios regarding the native population (supplementary table S2, Supplementary Material online). The first three models consider that ASIA was a single, panmictic population experiencing either 1) a constant population size, 2) exponential growth, or 3) instantaneous growth. Under these models, our sample from JP is considered as a representative sample from that population. Because our JP sample was obtained at a single location in JP and might not be representative of the ancestral range, however, we also considered two models where ASIA was represented by an unsampled population. In the first, we assumed that the JP population was recently colonized from ASIA and then experienced exponential growth. In the second, the native range was modeled as a structured population of a large collection of demes exchanging migrants at a constant rate (an island model), of which JP was a single deme and all nonsampled demes were represented by ASIA and modeled as a single population.

Using coalescent simulations, we assessed the ability of these models to reproduce the diversity patterns observed in our Japanese sample based on three statistics: average pairwise diversity (*π*), Tajima’s *D*, and the variance between sites in the number of observed alleles (*K*_sd_). We found that only the structured population model could reproduce the observed statistics both when considering all sites or noncoding sites only (supplementary fig. S7, Supplementary Material online). An ABC model choice analysis of the same data further supported the structured population model to be the most probable of the five tested (supplementary table S3, Supplementary Material online). Based on these results, we thus decided to represent the source population in our colonization scenarios by the structure model (fig. 4). We note that the strong negative Tajima’s *D* statistics observed at both coding and noncoding sites may in part also be the result of pervasive recent selection in these populations. In this case, we expect our model of population structure to still relatively accurately describe the diversity in the ASIA population, yet with a smaller migration rate than would be observed in nature.

#### ABC Analysis

We performed ABC analysis to infer the most likely model for the invasion history of *D. suzukii*. For this analysis, we used the six summary statistics with the highest power to distinguish between the three models tested (see Materials and Methods). Summary statistics were calculated using both coding and noncoding sites. Our analysis showed that Model 1 had the highest posterior probability (42%), although this was only marginally higher than the posterior probabilities of the other models (30% and 28%, table 3). Similar results were obtained when restricting the analysis to noncoding sites only (posterior probabilities 50%, 28%, and 22% for Models 1, 2, and 3, respectively).

**Table 3.** Model Choice Results.

Colonization History Models	Posterior Probability	Observed *P* Value	Tukey Depth	Tukey *P* Value
***1***	***0.4181***	***0.999***	***0.227***	***0.999***
2	0.2770	0.960	0.098	0.958
3	0.3049	0.943	0.105	0.937

Note.—Reported are the posterior probabilities for models of the colonization history of *Drosophila suzukii*. The model with the highest probability is shown in italic. The *P* value for the observed data and the Tukey depth and the *P* value for a Tukey test are reported.

To determine if our models can faithfully reproduce the observed data, we verified that the observed summary statistics fell well within the marginal distribution of summary statistics obtained through simulations (data not shown). However, because a potential mismatch may only manifest in higher dimensions, we also calculated two descriptive statistics among all summary statistics: the fraction of retained simulations with a smaller or equal marginal likelihood than the observed data (observed *P* value) and the fraction of retained simulations with a smaller or equal Tukey depth than the observed data (Tukey *P* value). We obtained high observed *P* value and Tukey *P* value for all three models, suggesting that they can reproduce the observed data in high-dimensional space (table 3).

**Fig. 5.**
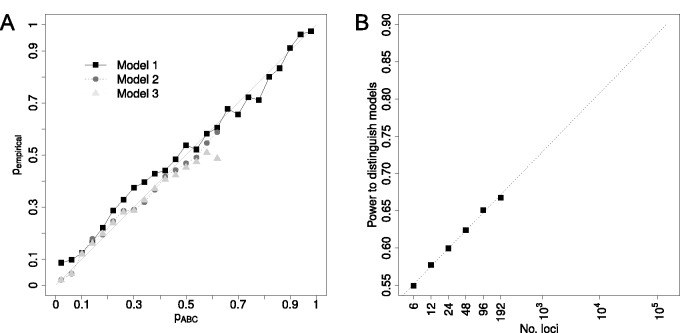
Validation of ABC model choice and power analysis. (*A*) A comparison of the posterior probability as estimated via ABC (*p*_ABC_) against an empirical estimate of the same probability obtained through simulations (*p*_empirical_, see Materials and Methods) reveals that our ABC approach for model choice is well calibrated. For both Models 2 and 3, however, only very few simulations resulted in high *p*_ABC_, which made the estimation of *p*_empirical_ problematic. We thus only plotted for bins for which we obtained at least 20 simulations (out of 10^5^ in total). (*B*) The relationship between number of loci and statistical power to distinguish the three colonization models for *D. suzukii*. Points denote results of simulations and line is the fitted linear regression. The *x*-axis (number of loci) is log_10_ scaled.

We next investigated whether our inferred posterior probability for a given model accurately reflects the probability to choose the true model. To do so, we generated 1,000 pseudo-observed data sets per model and calculated the posterior probability for each of these simulations using our ABC pipeline. We then plotted the estimated posterior probability against the fraction at which the chosen model corresponds to the true underlying model among all simulations in a particular bin of posterior probabilities (*p*_ABC_ vs. *p*_empirical_; fig. 5*A*). We found that *p*_ABC_ for Model 1 is well calibrated over the whole range. However, for Models 2 and 3, only very few simulations (<20) result in high *p*_ABC_ (fig. 5*A*) making the estimation of *p*_empirical_ problematic and suggesting that Models 2 and 3 are hard to distinguish.

#### Parameter Estimates from ABC Analysis

**Fig. 6.**
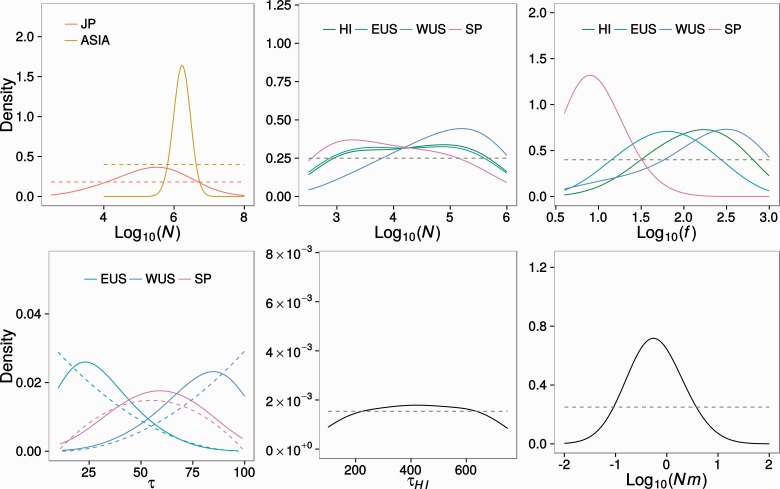
The posterior probability of the parameters of the three colonization models for *D. suzukii* weighted by the posterior probability of each model, where *N* is the current population size, *f* is the number of founding individuals, *τ* is the colonization time for each population (in generations), and Nm is the migration rate among demes in the structured Japan population model. Note that the posterior distribution of *τ*_HI_ was plotted separately from the remaining *τ* estimates due to its unique prior range.

Because the model choice analysis did not produce a high posterior probability for any of the three models and given that parameters are shared among the three models, we weighted the posterior probability of the parameters by the posterior probability of each model (table 4). This procedure allowed us to obtain parameter estimates while accounting for the uncertainty in choosing the correct model. Marginal posterior distributions are shown in figure 6, and characteristics of these distributions are given in table 4.

**Table 4.** Priors and Weighted Posterior Estimates for Parameters of the Three Models of Colonization for *Drosophila suzukii*.

Parameter	Population (*i*)	Prior	Mode	Mean	Median	Q5%	Q95%
Log_10_(*N_i_*)	Asia	*U*[4, 8]^a^	6.27	6.26	6.26	5.94	6.56
	Japan (JP)	*U*[2, 6]^a^	5.64	5.31	5.40	3.46	6.88
	Hawaii (HI)		5.18	4.32	4.37	2.78	5.77
	Western United States (WUS)		5.37	4.66	4.77	3.12	5.84
	Eastern United States		3.66	4.25	4.24	2.76	5.74
	Spain		3.03	4.04	3.98	2.68	5.61

Log_10_(*f_i_*)	Hawaii	*U*[0.6, 3]^a^	2.30	2.10	2.14	1.23	2.84
	Western United States		2.52	2.14	2.25	1.00	2.89
	Eastern United States (EUS)		1.81	1.79	1.79	0.95	2.63
	Spain (SP)		0.87	1.03	0.98	0.65	1.56

*τ_i_*	Hawaii (HI)	*U*[100, 750]^a^	421	431	434	147	704
	Western United States (WUS)	*U*[10, 100]^b^ *τ*_HI_ > *τ*_WUS_ > *τ*_SP_ > *τ*_EUS_	88	75	78	42	97
	Eastern United States (EUS)		21	32	29	12	62
	Spain (SP)		60	58	58	24	90

Log_10_(Nm)	—	*U*[−2, 2]^a^	−0.27	−0.21	−0.23	−1.05	0.69

*μ* × 10^9^	—	*N*(3.46, 0.28)^c^	3.49	3.49	3.49	2.46	4.52

Note.—The models share the same parameters (population sizes *N*, number of founder *f*, time of colonization *τ*, migration rate Nm, and mutation rate *μ*) and the posterior estimates are weighted by the posterior probability of each model.

^a^*U*[*x*, *y*]: uniform distribution between *x* and *y*.

^b^*U*[10, 100] on each *τ_i_* initially, but only combinations in line with the stated rules were accepted.

^c^*N*(*x*, *y*): normal distribution with mean *x* and standard deviation *y*.

The median estimate of the population sizes *N* of the sampled population in JP was 1 order of magnitude smaller than *N* of ASIA (∼2.5 × 10^5^ vs. ∼2.5 × 10^6^, respectively, table 4). However, we note that the posterior distributions for *N* of JP and all colonized populations (HI, WUS, EUS, SP) were quite similar to the prior (fig. 6). Although the number of founders (*f*) for SP was estimated to be extremely low (∼10), *f* was estimated to be substantially higher (∼100) for the rest of the colonized populations (HI, WUS, EUS). These results are consistent with our observation of a major reduction in *π* for SP, indicating a strong bottleneck at the time of colonization for this population. However, the posterior distributions for the colonization times (*τ*) were almost identical to the prior (fig. 6), indicating that there is almost no information on the data about these parameters. Finally, we estimated a migration rate Nm of ∼0.6 between JP and ASIA (fig. 6). This estimate corresponds to an Fst >0.4 when assuming an infinite island model (Wright 1943), which suggests substantial population structure in *D. suzukii* populations in Asia.

Although these results were obtained using both coding and noncoding sites, multiple lines of evidence suggest that these inferences are not likely to be strongly compromised by selection. First, we obtained almost identical results when restricting the analysis to noncoding sites only (supplementary fig. S9, Supplementary Material online). Second, selection is expected to be weak in recently colonized populations, as they have undergone a bottleneck during colonization followed by rapid population growth. Finally, and as outlined above, we expect the demographic model to accurately describe the genetic diversity in the native range even under selection, but we note that we overestimate structure (i.e., underestimate Nm) if selection was acting strongly on the loci studied here.

We next tested for biased posteriors for the parameter estimates. To do so, we generated 1,000 pseudo-observed data sets for each model by sampling the parameters from their inferred marginal posterior distributions. The position of the true parameters within the posterior distribution (posterior quantiles) must reflect the probability denoted by the posterior distribution. We examined the distributions of posterior quantiles of all the parameters for each model, which should be uniformly distributed if the posteriors are unbiased. We found this to be the case for most parameters, except for the population size of ASIA, the mutation rate, and the colonization times of EUS and WUS populations (*N*_ASIA_, *μ*, *τ*_EUS_, and *τ*_WUS_; supplementary fig. S10, Supplementary Material online). The small biases that we obtained for *μ* and the two colonization times are likely due to the highly informative priors (e.g., the experimentally measured *μ* for *D. melanogaster*). The smallest high posterior density interval containing the true parameter is an alternative descriptive statistic to validate marginal posterior distributions and should also be uniform across replicates. Based on this metric, we found that all parameter estimates were unbiased, again with the exception of *μ* (supplementary fig. S11, Supplementary Material online).

### Power to Infer Colonization Histories: Contrasting Old and Recent Invasions

Because the invasion of *D. suzukii* has occurred only very recently, it is likely that the introduced populations are still far from a mutation–drift equilibrium and hence may lack sufficient diversity to infer the number of founders, the time of invasions, or even to detect population structure within the newly colonized range from our data set (Fitzpatrick et al. 2012). Indeed, when inferring the parameters of the recent spatial expansion of the introduced cane toad in northern Australia, historical records were much more informative than genetic data (Estoup et al. 2010). We thus explored both the specific question of power to infer colonization history of *D. suzukii* and the general question of how much genetic data are required to infer colonization histories with sufficient power.

We first inferred the power to distinguish the three models of invasion of *D. suzukii* as a function of the number of loci sequenced, assuming that each locus is independent and 450 bp long (to mimic our experimental study). For the six loci used in this study, our power to infer the correct model is limited to only 55%. We further found that the increase in power with more loci sequenced is well-described as a logarithmic function up to the largest number loci tested (192, fig. 5*B*). Using this function, we predict that more than 10^5^ independent loci, corresponding to at least 45 Mb of sequence, would be needed to have 90% power to distinguish between the three models. These results are robust to the number of ABC simulations performed (10^4^, 10^5^, and 10^6^ simulations give very similar prediction of power versus number of loci sequenced; supplementary fig. S8, Supplementary Material online). The results of this power analysis apply more generally to recently invasive species and thus indicate that in general, distinguishing alternative models of very recent invasions remains challenging even with full genome data.

Given that the weighted posterior estimates of *τ* are almost identical to our prior expectation (fig. 6), we next investigated what kind of information would be needed to infer these parameters accurately. We explore this question in general terms, as a function of two critical parameters: the number of founders and the time of colonization. However, we kept the current population size constant at 10,000 individuals.

**Fig. 7.**
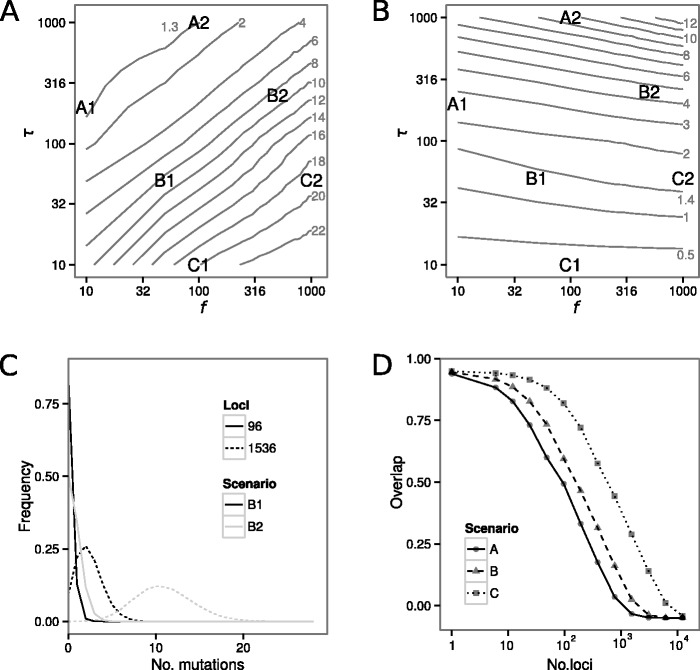
Simulation results for a population experiencing exponential growth that started at time *τ* generations ago with *f* founders and the number of loci needed to infer those parameters. (*A*) The expected number of ancestral lineages *L*_A_. Note that the expected number of ancestral lineages is identical for many combinations of *f* and *τ*, as is indicated by the isolines*.* (*B*) The expected total length of the genealogy since colonization *G*_L_ for different combinations of *τ* and *f*. Note that *G*_L_ is also identical for many combinations of *f* and *τ*. In panels *A* and *B*, we highlight pairs of parameter combinations (A1 and A2, B1 and B2, and C1 and C2)—these pairs of parameter combinations have the same number of ancestral lineages *L*_A_ but different colonization times and numbers of founders (see panel *A*). Panel *C* depicts the probability distribution of observing a specific number of new mutations since colonization for parameter combinations B1 and B2 (black and gray lines, respectively) calculated for 96 and 1536 sequenced loci (solid and dashed lines, respectively). Panel *D* shows the overlap in such distributions between our scenarios with the same expected number of ancestral lineages (A1 vs. A2, B1 vs. B2, and C1 vs. C2, see panel *A*) for an increasing number of loci.

In recent invasions, most of the diversity in the colonized population comes from the source population, which is then subjected to drift. One way to quantify the diversity that comes from the source population is to calculate the number of lineages in a sample that have not coalesced at the time of colonization *τ,* a quantity we will term “number of ancestral lineages” and denote here by *L*_A_. Interestingly, the expected number of ancestral lineages *E*(*L*_A_) is identical for several combinations of *f* and *τ* (fig. 7*A*)*.* In general terms, these results imply that it is impossible to distinguish between a recent colonization with few founders and an older colonization with many founders based on the diversity from source populations only. Distinguishing between these alternatives requires knowing one of the two parameters by independent means, such as historical records available for *D. suzukii* which inform *τ.*

If neither *f* nor *τ* are known, the joint inference of *f* and *τ* will rely on new diversity generated in the colonized population. One way to characterize new diversity is to calculate the length of the genealogy since colonization (the sum of all branch lengths since *τ*), which is proportional to the expected number of new mutations. Here, we will term this quantity “genealogy length” and denote by *G*_L_. Although we again find the same expected genealogy length *E*(*G*_L_) for several combinations of *f* and *τ* (fig. 7*B*)*,* it appears that new mutations provide information about *τ* and *f* that is complementary to the information obtained from the diversity that comes from the source populations (almost perpendicular isolines in fig. 7*A* and *B*). This result suggests that each parameter combination of *f* and *τ* can be uniquely identified based on the combination of ancestral and novel diversity, given that enough polymorphisms have been surveyed.

We next investigated the number of loci required to obtain enough new mutations such that scenarios with the same expected diversity from the source populations can be distinguished. To do so, we focused on three comparisons (A, B, and C) between two scenarios (1 and 2) with the same expected number of ancestral lineages *E*(*L*_A_). We then use these scenarios to explore the general power to infer colonization scenarios as a function of the amount of genetic data. Among these, scenarios A1 and A2 have the smallest, scenarios B1 and B2 have an intermediate, and scenarios C1 and C2 have the highest expected number of ancestral lineages (fig. 7*A*).

For each scenario, we first obtained the probability distribution of observing a specific number of new mutations as a function of sequenced loci and assuming the experimentally inferred mutation rate for *D. melanogaster* (Keightley et al. 2009). The overlap in these distributions between two scenarios is then a measure of how well they can be distinguished from each other based on new diversity. To illustrate our approach, we first show such distributions for the two scenarios, B1 and B2 (for respectively 96 and 1536 loci) in figure 7*C*. As is indicated by the large overlap for a sequencing effort of 96 loci, it is impossible to distinguish these scenarios as both likely result in very similar numbers of new mutations (fig. 7*C*). In contrast, for a larger sequencing effort of more than 1,000 loci, the overlap becomes much smaller, possibly enabling distinguishing the two scenarios (fig. 7*C*).

In figure 7*D*, we then show the obtained overlap in the probability distribution of observing a specific number of new mutations between the scenarios A1 and A2, B1 and B2, as well as C1 and C2 for a wide range of number of sequenced loci. Our simulations revealed that 10^3^–10^4^ loci would be needed to distinguish quite distinct scenarios of *τ* and *f* (fig. 7*D*). To distinguish combinations of *τ* and *f* that are closer in parameter space than in the scenarios that we investigated, we would need an even larger sequencing effort. However, we note that the required number of loci is of course proportional to the mutation rate such that using loci with a higher mutation rate (e.g., microsatellites) would reduce the required number of loci. In addition, because recombination rates often exceed rates of mutation, incorporating patterns of linkage disequilibrium into the analysis may also reduce the required number of loci. However, we note that recent recombination events can only be detected accurately if sufficient genetic diversity is present.

We finally investigated how larger sample sizes might help in jointly inferring *f* and *τ*. A classic result of coalescent theory is that the expected number of mutations among *n* samples in a constant population is proportional to

$$\sum_{j=1}^{n-1}\frac{1}{j},$$

implying that doubling the sample size from 25 to 50 increases the expected number of mutations by only 18.6%. However, in the case of exponential growth, this increase is likely much higher. Indeed, using our simulation framework, we found that in recently colonized and exponentially expanding populations, doubling the sample size increases the expected number of mutations between 40% and 90% for parameters relevant for *D. suzukii* (supplementary table S4, Supplementary Material online). These results suggest, in line with findings under constant-sized populations, that using large numbers of loci is generally more cost effective than using large sample sizes.

## Conclusions and Future Directions

Our analysis provides insight into the colonization history of Europe and the continental United States by *D. suzukii*. We find limited population structure within the continental United States, which may be due to the recency of this invasion. However, this limited structure could be due in part to gene flow among populations and/or recurrent invasions. This would be discouraging for potential management strategies in the continental United States, as it suggests that local eradication efforts may have limited success. Our haplotype-sharing data are suggestive that the recent colonization of the continental United States and Europe are separate demographic events. However, we are unable to resolve the colonization history of this species cleanly due to the recency of invasions and the sub-genome-level sampling conducted here.

In moving forward, it is clear that dense, worldwide sampling particularly in the ancestral range of this species will be needed to identify source population(s) of both the North American and European invasions. Our ABC analysis indicates that genome-level data will be required to distinguish among alternative colonization models and to jointly infer the number of founding lineages and colonization times with any precision. These population genomic data may be valuable for control efforts, as these source populations could potentially be targeted to limit future colonization opportunities. Moreover, repeated sampling over time from recently colonized areas will be required to adequately describe patterns of gene flow, determine whether recurrent invasions are in fact occurring, and refine our understanding of the mechanisms mediating the dispersal of this species.

In a broader context, our data and analysis show that the reconstruction of very recent invasions, such as that of *D. suzukii*, from genetic data requires an enormous number of loci, which even full genomes may be unable to provide. We further show that inferring the timing of an invasion jointly with the number of founding individuals is possible, but requires potentially thousands of loci. In contrast, even a small set of sequence loci allowed us to characterize the genetic diversity in the native range of the species and to quantify the general loss in genetic diversity following recent introductions.

## Materials and Methods

### Sampling Specimens

*Drosophila suzukii* males were collected between the months of August 2011 and February 2012 from 12 natural populations from around the world (fig. 1). Our sample locations were the following: Ehime, Japan (JP); Na Pali-Kona Forest Reserve, Kauai, Hawaii (HI); Stanford, California (ST), California; San Diego, California (SD), California; Fenville, Michigan (MI); Pepperell, Massachusetts (MA); Martinsburg, West Virginia (WV); Laurel Springs, North Carolina (NC); Savannah River Site, South Carolina (SC); Atlanta, Georgia (GA); Wimauma, Florida (FL); and Barcelona, Spain (SP). Flies were aspirated, live trapped, or passively collected using hanging traps containing a solution of propylene glycol and red wine (13% red wine by volume). Time-course assays suggest reliable PCR amplification of mitochondrial DNA following up to 8 days of immersion in this solution, and reliable PCR amplification of nuclear markers following 2–5 days of immersion (data not shown). Between 7 and 24 males were analyzed from each location. The species identity of these males was confirmed by visualization under a dissecting microscope, and all specimens were stored in 95% ethanol until use.

### Genomic DNA Extraction

Genomic DNA was extracted from 246 single males from the 12 populations listed above. Flies were soaked in deionized water and air dried prior to DNA extraction. Individual flies were homogenized with a motor-driven pestle in 1.7 ml tubes containing a cell lysis solution of 98.7 µl cell lysis buffer (pH 8; 10 mM Tris–HCl, 100 mM ethylenediaminetetraacetic acid, 2% sodium dodecyl sulfate) and 1.3 µl proteinase K (200 µl/ml). This was incubated at 65 °C for 15 min, and then cooled to room temperature (RT). RNase treatment was completed by the addition of 2 µl RNase A (1 mg/ml). The solution was mixed by inverting the tubes and was subsequently incubated at 37 °C for 40 min before being cooled to RT. Proteins were precipitated by the addition of 33 µl of 7.5 M NH_4_Ac. Samples were vortexed for 10 s and chilled on ice for 5 min before being centrifuged at 23,000 relative centrifugal force (RCF) for 3 min. The supernatant was transferred to new 1.7 ml tubes containing 100 µl isopropanol and subsequently mixed by inverting the tubes. Tubes were then centrifuged at 23,000 RCF for 5 min, and then the supernatant was discarded. The remaining DNA pellet was rinsed with 100 µl of 70% ethanol and centrifuged at 23,000 RCF for 1 min. The tube was then inverted and allowed to dry for 30 min, before an overnight resuspension in 25 µl deionized water.

### Locus Choice

We designed primers to six putatively X-linked regions in *D. suzukii*. To identify these loci, we restricted ourselves to those genes with 1:1 orthologs in the *D. melanogaster* species group (Larracuente et al. 2008). We also restricted ourselves to genes that are X-linked in the 12 sequenced *Drosophila* genomes (Bhutkar et al. 2008). Using the Flybase annotations of the *D. melanogaster* genome, we further restricted ourselves to intron-containing genes in the hopes that we could include both coding and noncoding sequences in our analysis (although note that one locus, 30437, is entirely noncoding). This was motivated by estimates of polymorphism from other species which are often reported for coding sequences and/or noncoding sequences; for ease of comparison to previous work, we wished to include both types of sites in our analysis. We BLASTed (Altschul et al. 1997) the *D. melanogaster* gene sequences of these X-linked intron-containing 1:1 orthologs against a draft assembly of the *D. suzukii* genome (Eisen MB, personal communication) to find orthologous sequences in *D. suzukii*. We used several criteria to finalize our locus choice. First, we required that the *D. melanogaster* query only had significant homology to one continuous region of a single *D. suzukii* scaffold. Second, we spaced our loci across the (*D. melanogaster*) X chromosome to minimize linkage across loci. Third, we prioritized candidate loci based on the quality and length of the pairwise BLAST alignments. Finally, we sought loci in which we could anchor our primers in exons and generate amplicons of 600–800 bp. This approach yielded 11 candidate loci. Primers for six of these loci worked exceptionally well and we chose those for our analyses. These loci correspond to fragments of the presumed orthologs of *D. melanogaster* FBgn0029789, FBgn0029997, FBgn0026206, FBgn0017651, FBgn0001083, and FBgn0030437. The physical positions of these genes in *D. melanogaster* are respectively 5.6, 8.0, 9.0, 10.7, 11.9, and 12.9 Mb. Our naming scheme for our loci was based on the *D. melanogaster* gene names and the loci are respectively named 29789, 29997, 26206, 17561, 1083, and 30437. Primer sequences are presented in supplementary table S1, Supplementary Material online. We note that the genome of *D. suzukii* has recently been published (Chiu et al. 2013); in all cases, BLASTing our sequence fragments against the published genome reveals a single, high quality, continuous alignment over the entire length of each of our loci. The *D. suzukii* genome has not as yet assembled into chromosomes, so we cannot determine the precise genomic location of the loci studied here.

### PCR Amplification and Sequencing

All PCR reactions were completed using 8 µl reaction volumes that each contained 4 µl of 2× Qiagen PCR MasterMix, 0.2 µl of each 10 µM primer, 2.6 µl ddH_2_O, and 1 µl genomic DNA. The PCR amplification conditions used for all samples were 94 °C for 3 min, 35 cycles of 94 °C for 30 s, 56 °C for 1 min, 72 °C for 1 min, and a final extension of 72 °C for 7 min. PCR reactions were enzymatically cleaned using the Affymetrix ExoSAP-IT PCR cleanup reagent. The enzymatically cleaned reactions were prepared for sequencing with an Applied Biosystems BigDye Terminator v3.1 cycle sequencing kit using 0.5 µl BigDye Terminator v3.1, 1.75 µl 5× sequencing buffer, 0.3 µl of 10 µM primer, 4.45 µl of ddH_2_O, and 3µl of cleaned PCR product. These reactions were sequenced on an Applied Biosystems 3730xl DNA Analyzer.

### Data Analysis

Sequences were manually aligned in Sequencher 5.0. Gaps are produced in the alignment procedure and represent insertions or deletions. These sequences are available at GenBank (accession numbers KM607862-KM609015). Our analyses required ungapped data and we thus removed sites for which one or more individuals had gaps (removed a total of 452 bp). We concatenated the sequences of all loci, which resulted in an ungapped data set of 2,745 bp of 246 individuals from 12 populations. However, this data set still contained 21.4% missing data (positions at which we failed to determine the genotype of an individual). Because most of our analyses can account for missing data, we did not attempt to remove it from the alignment.

Haplotype diversity was estimated in DnaSP v5 (Librado and Rozas 2009). We estimate the reduction in haplotype diversity at a particular locus in a particular population relative to JP (as a proxy for an ancestral sample) as follows:

$$\frac{{\text{Haplotype Diversity}}_{\text{Sample}}-\text{​}{\text{Haplotype Diversity}}_{\text{Japan}}}{{\text{Haplotype Diversity}}_{\text{Japan}}}.$$

When estimating the proportional reduction in haplotype diversity in each population overall, we calculate the percent change at each locus as mentioned earlier, then take an average across loci weighted by locus size. Haplotype networks were constructed in TCS 1.21 (Clement et al. 2000) excluding gaps.

Nucleotide diversity (*π*) for each population was calculated for all sites and noncoding sites only following a previous example to account for missing data (Hutter et al. 2006). Standard error for *π* was calculated by bootstrapping by site. We estimate the percent change in *π* in each derived population relative to the Japanese population as follows:

$$\frac{\pi_{\text{sample}}-\pi_{\text{Japan}}}{\pi_{\text{Japan}}}.$$

A pairwise paired two-tailed bootstrap test was performed to assess significance for the difference in *π* between the derived populations and JP.

We also performed analysis of population structure using the program *ADMIXTURE* (Alexander et al. 2009). To run *ADMIXTURE*, we converted our data set into PED files using a custom Perl script and then we converted the PED files into BED files using the program PLINK (Purcell et al. 2007). We ran *ADMIXTURE* using the default parameters for *K* values 2–6. We then used a custom *R* script to plot the maximum-likelihood estimates of the parameter matrix *Q* of admixture coefficients obtained with *ADMIXTURE*.

Furthermore, we conducted an AMOVA using Arlequin 3.5 (Excoffier and Lischer 2010) to obtain pairwise Fst between all pairs of populations. These calculations were restricted to all sites for which at least two individuals per population included in the calculation had data. We then used permutations to assess statistical significance of the Fst values (1,000 permutations).

Finally, we refined our focus further to the seven populations in the EUS for two additional tests. We first conducted an AMOVA using Arlequin 3.5 (Excoffier and Lischer 2010) to estimate within and between population variance components. Second, we conducted a Mantel test for these seven populations using isolation-by-distance web service (http://ibdws.sdsu.edu/∼ibdws/; Jensen et al. 2005). Geographic distances were estimated using the Geographic Distance Matrix Generator (http://biodiversityinformatics.amnh.org/open_source/gdmg; Ersts [Internet]) based on latitude and longitude co-ordinates of the collection sites. Genetic distances were estimated using pairwise Fst (see above). As two alternate measures of genetic distance for the purposes of the Mantel test, we (minus) log-transformed Fst and also used Fst/(1 − Fst) (Rousset 1997).

### Approximate Bayesian Computation

We used an ABC approach (Wegmann et al. 2009, 2010) to compare five different demographic models for the Japanese population and three alternative models for the colonization history of *D. suzukii*. We considered the following models for the Japanese population: constant population, exponential growth, instantaneous growth, exponential growth following colonization from another unsampled population, and structured population. For the structured population model, we assumed an island model where our sample comes from one of the “islands” connected to the unsampled continent via migration.

To formulate models for the colonization history of *D. suzukii*, we grouped the individuals sampled in different locations into five distinct geographical areas: JP, HI, WUS (SD and ST), EUS (all remaining continental US samples), and SP. These groupings were based on the demographic history of this species. In particular, we separated the WUS and EUS because *D. suzukii* appeared in the West at least 1–2 years before appearing in the east (Hauser 2011) and we wanted to test the hypotheses that these invasions were independent events. We used the taxonomic records of each region as a guideline to establish the different colonization times.

### ABC Pipeline

We generated 10^5^ simulations for each model using fastsimcoal (Excoffier and Foll 2011). We assumed that loci were independent and that there was no recombination within loci. We believe that this assumption is reasonable given the short length of our loci and the recency of the demographic events that are the focus of this study. The priors for the different neutral demographic models for the Japanese population and the invasion history of *D. suzukii* are given in supplementary table S2, Supplementary Material online and table 4, respectively. Our prior for the mutation rate per generation (*μ*) was a normal distribution with mean 3.46 × 10^−9^ and variance 0.28 × 10^−9^, corresponding to the experimental estimate for *D. melanogaster* and its associated uncertainty from the literature (Keightley et al. 2009). The standard deviation was chosen to encompass a 2-fold wider range than the 95% confidence intervals reported in the Keightley et al. (2009) study. We used a uniform prior for the colonization times of the different continents, but constrained the relative colonization times based on historical records. Specifically, we required that the colonization time of WUS had to precede the colonization time of SP and the colonization time of SP had to precede the colonization time of EUS.

Choosing appropriate summary statistics is an important step for ABC. However, choosing the relevant statistics for ABC remains difficult with a few summary statistics likely lacking necessary information and a large set likely containing many potentially correlated summary statistics that contribute noninformative noise (Beaumont et al. 2002; Wegmann et al. 2009). This is particularly true for model choice, where even statistics sufficient for all models may lead to biased inference (Robert et al. 2011).

We computed an initial set of 67 summary statistics for simulated and observed data sets using Arlequin v.3.5.1.3. For each population, these summary statistics included the number of segregating sites (*S*), the average pairwise diversity (*π*), the number of private segregating sites (pr*S*), the standard deviation of the number of alleles and heterozygosity between sites (*K*_sd_ and *H*_sd_, respectively), Tajima’s *D*, the logarithm of the ratio of *S* in each colonized population over *S* in JP (log(*S_i_*/*S*_JP_), *i* = HI, WUS, EUS, SP), and the ratio pr*S*/*S* for WUS and SP. We also used the mean and standard deviation of *S* and *π* over all populations and the pairwise diversity and pairwise Fst between populations.

For the comparison of the five demographic models for the Japanese population, we used three statistics: *π*, Tajima’s *D*, and *K*_sd_. For the comparison of the three models for the invasion history of *D. suzukii*, we calculated the power of combinations of statistics to distinguish the alternative models (those combinations were created from the initial set of 67 statistics). We followed Chu et al. (2013) and identified appropriate combinations of summary statistics based on empirical power assessment. Briefly, we calculated the power to choose the correct model by ABC given all pairwise combinations of the initial set of statistics and iteratively added additional statistic until no improvement in the power is observed. We then chose the combination of statistics with highest power for all our model comparisons, which were Fst_SP-EUS_, Fst_WUS-EUS_, Fst_HI-EUS_, Fst_HI-SP_, pr*S*_WUS_/*S*_WUS_, and pr*S*_EUS_/*S*_EUS_. With those six statistics we had power of 55.7% to discriminate among the three models, compared with 43.9% when using all statistics. As was shown in a recent study, the low power obtained with all statistics is expected in a large set of summary statistics due to difference between models in the proportionality constant between the full likelihood and the likelihood of the summary statistics (Robert et al. 2011),

To estimate the posterior densities for the parameters, we used a partial least squares (PLS) approach implemented in ABCtoolbox, instead of using the actual summary statistics. With the PLS approach, we can find sets of linear combinations of statistics that best explain the variance in the model parameter space. We found that seven PLS components were sufficient to explain the variance of the parameters. Note that we cannot use this approach for model choice because the PLS transformation to the statistics is done for each model separately (for model choice the summary statistics must be the same between the compared models). To infer the posterior probability of each model and of the parameters, we retained 10^3^ simulations that produced statistics closest to the observed data and fitted a general linear model (GLM) to those following previous example (Leuenberger and Wegmann 2010).

### ABC Validation

To validate our model choice results, we computed two statistics: the observed *P* value and the Tukey *P* value. To calculate the observed *P* value, we compared the marginal likelihood of the observed data against the marginal likelihood of a set of retained simulations (these produced statistics that are close to the observed data). The observed *P* value is then the fraction of retained simulations with a smaller or equal marginal likelihood than the observed data. A low *P* value suggests that the model that was used to generate the data in the simulations does not fit well the observed data.

The Tukey depth (or Tukey half-space depth; Cuesta-Albertos and Nieto-Reyes 2008) quantifies how central a point is among a set of points in high-dimensional space. For example, in one dimension the median has a Tukey depth of 0.5. The Tukey depth of a chosen simulation (or the observed data) is defined as the smallest fraction of retained simulations which can be separated from the rest of the simulations using a hyper plane through the chosen simulation (or the observed data). A small Tukey depth thus indicates that the chosen simulation is far away from the center of all points. We compared the Tukey depth of the observed data against the Tukey depth of a set of retained simulations to compute a *P value*, defined as the fraction of retained simulations with a Tukey depth smaller than that of the observed data.

### Coalescent Simulations

The present diversity observed in a population that has been recently founded is the sum of diversity coming from the source population and the diversity generated from new mutations since the colonization event. To distinguish between these two, we inferred through simulations both the expected number of lineages (Lc) that have not coalesced until the time of colonization, as well as the expected length of the genealogy of the sample since colonization. These quantities were estimated as the means across 1,000 simulations of coalescent times in an exponentially growing population with present population size of 10,000 individuals and for various numbers of founding individuals and colonization times following the approach of Slatkin and Hudson (1991), as modified by Nielsen (2000). To obtain the distribution of mutations that arose since the colonization event for *l* loci, we simulated the total length of *l* genealogies and sprinkled mutations on each of them assuming a Poisson distribution with rate *μL*, where *μ* is the mutation rate as experimentally measured in *D. melanogaster* (Keightley et al. 2009) and *L* is the length of the simulated loci. Except where stated otherwise, all simulations were conducted for a sample size of 25 and with a mutation rate of 3.46 × 10^−9^, corresponding to the experimental estimate for *D. melanogaster* (Keightley et al. 2009).

## Supplementary Material

Supplementary figures S1–S11 and tables S1–S4 are available at *Molecular Biology and Evolution* online (http://www.mbe.oxfordjournals.org/).

Supplementary Data
