# Supplementary material for: Drosophila suzukii: The Genetic Footprint of a Recent, Worldwide Invasion
Source: Mol Biol Evol. 2014 Aug 25;31(12):3148–63. doi: 10.1093/molbev/msu246 (PMC4245814; doi:10.1093/molbev/msu246)
Supplement: Supplementary Data [file supp_31_12_3148__index.html]

Drosophila suzukii: the genetic footprint of a recent, world-wide invasion — Drosophila suzukii: The Genetic Footprint of a Recent, Worldwide Invasion — Drosophila suzukii: The Genetic Footprint of a Recent, Worldwide Invasion — Supplementary Data 

# *Drosophila suzukii*: The Genetic Footprint of a Recent, Worldwide Invasion

## Supplementary Data

files

**Files in this Data Supplement:**

- Supplementary Data - pdf file
