# Supplementary material for: Drosophila suzukii: The Genetic Footprint of a Recent, Worldwide Invasion
Source: Mol Biol Evol. 2014 Aug 25;31(12):3148–63. doi: 10.1093/molbev/msu246 (PMC4245814; doi:10.1093/molbev/msu246)
Supplement: Supplementary Data [file supp_msu246_SupplementaryFiguresAndTables.pdf]

Supplementary Figure 1

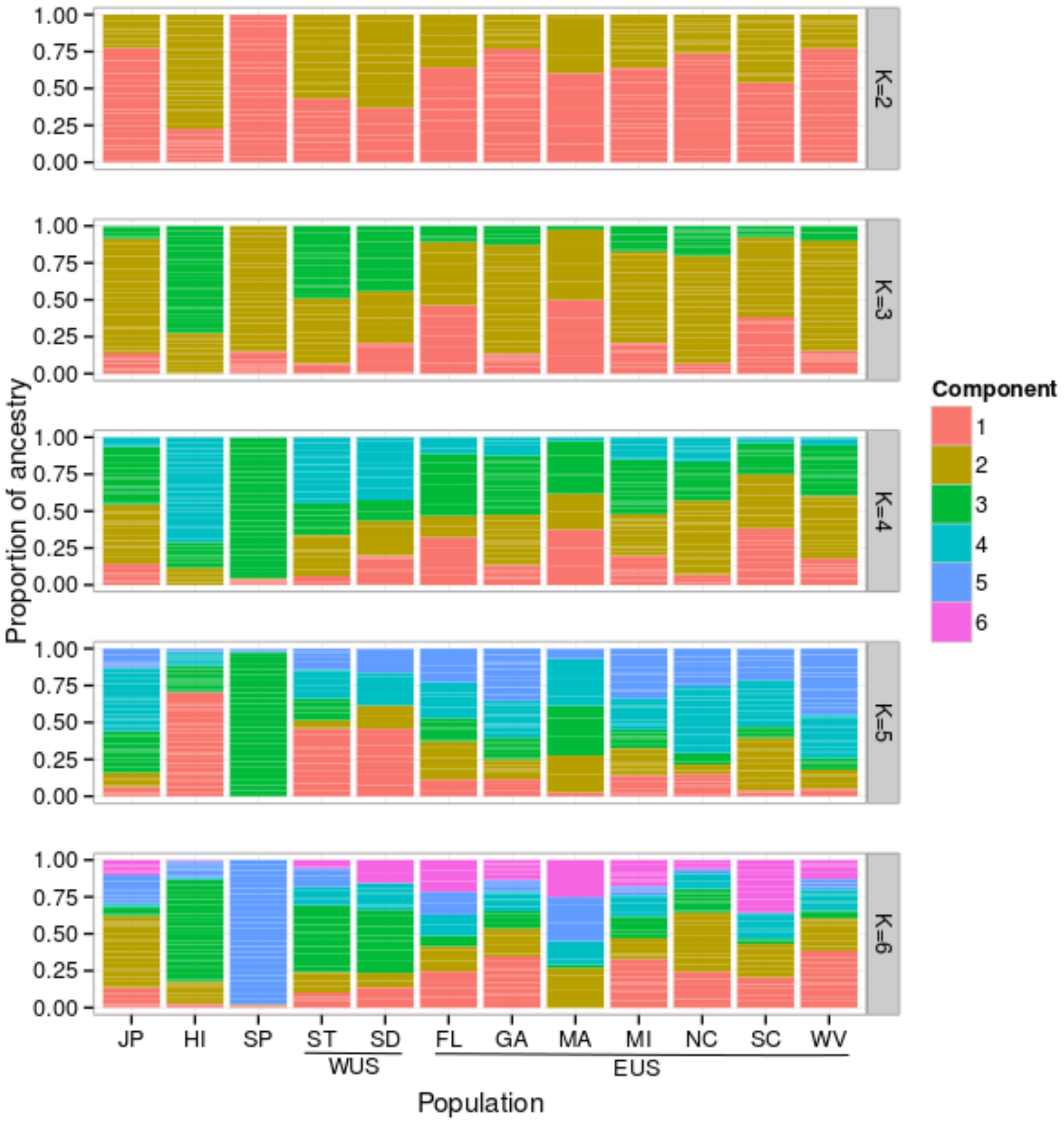

Supplementary Figure 1. Population structure of the 12 *D. suzukii* populations. Each population is represented by a vertical stacked column consisting of the proportions of genetic components (K=2-6).

Supplementary Figure 2

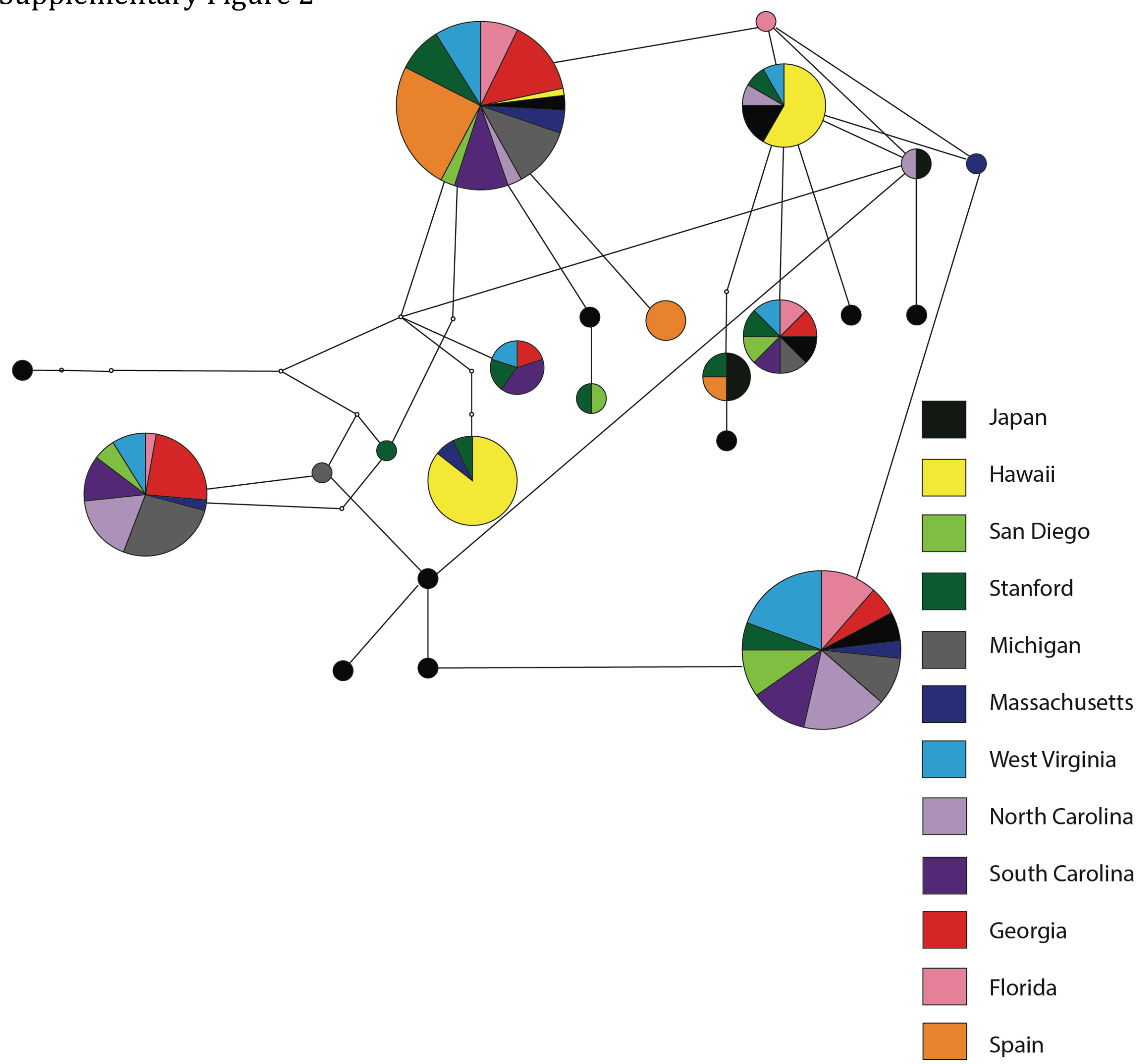

Supplementary Figure 2: Haplotype network for locus 29789

Supplementary Figure 3

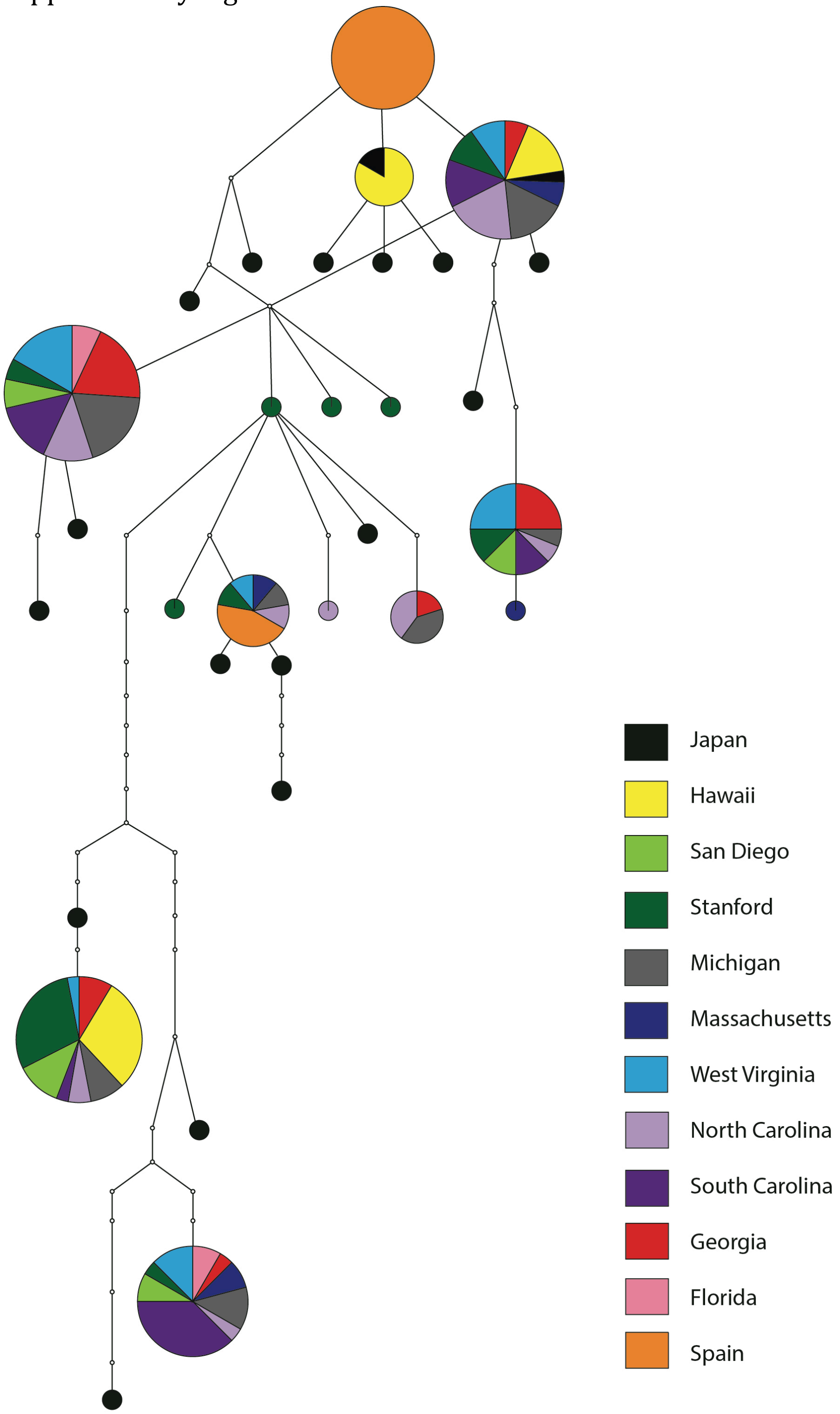

Supplementary Figure 3: Haplotype network for locus 29997

Supplementary Figure 4

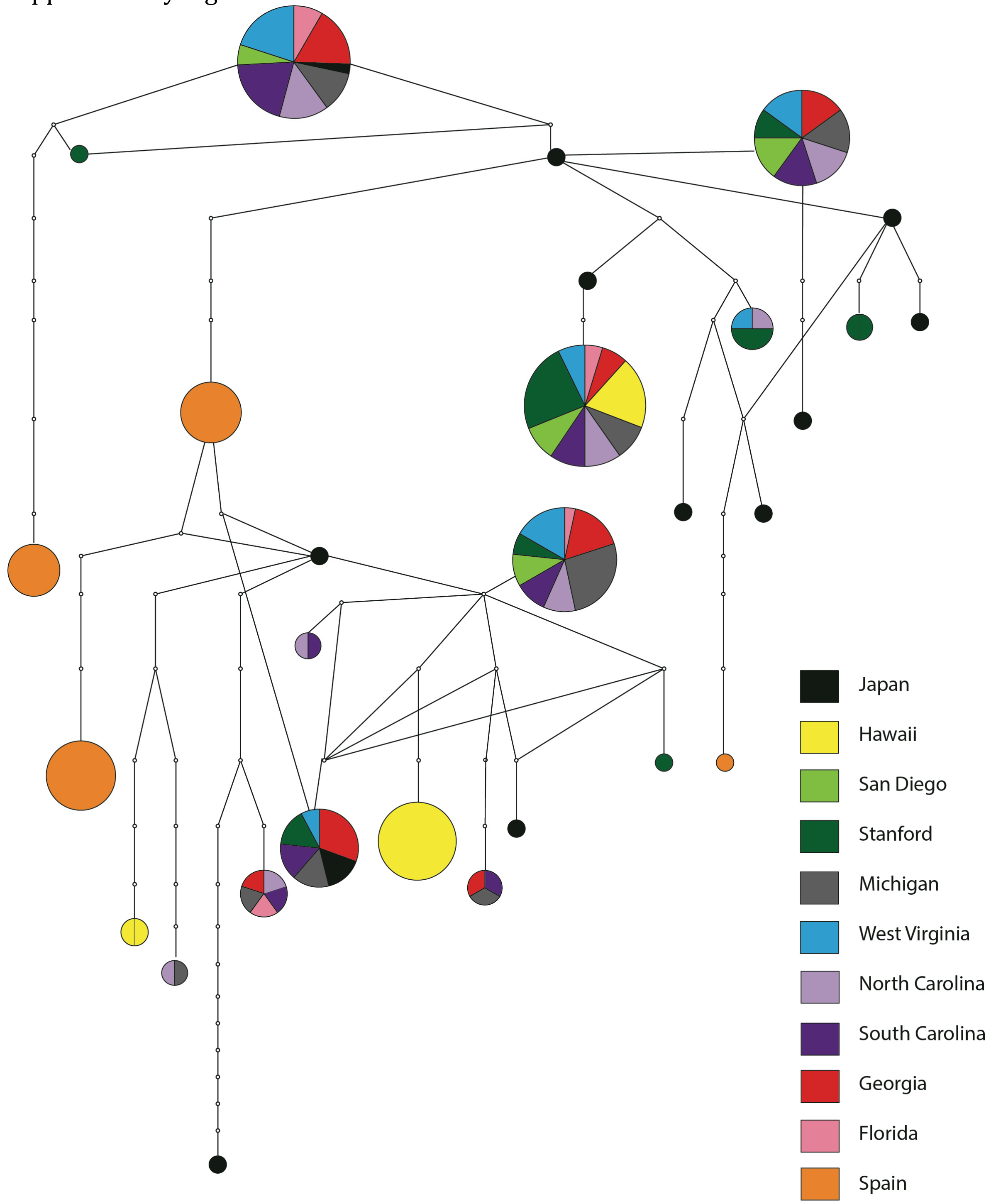

Supplementary Figure 4: Haplotype network for locus 17561

Supplementary Figure 5

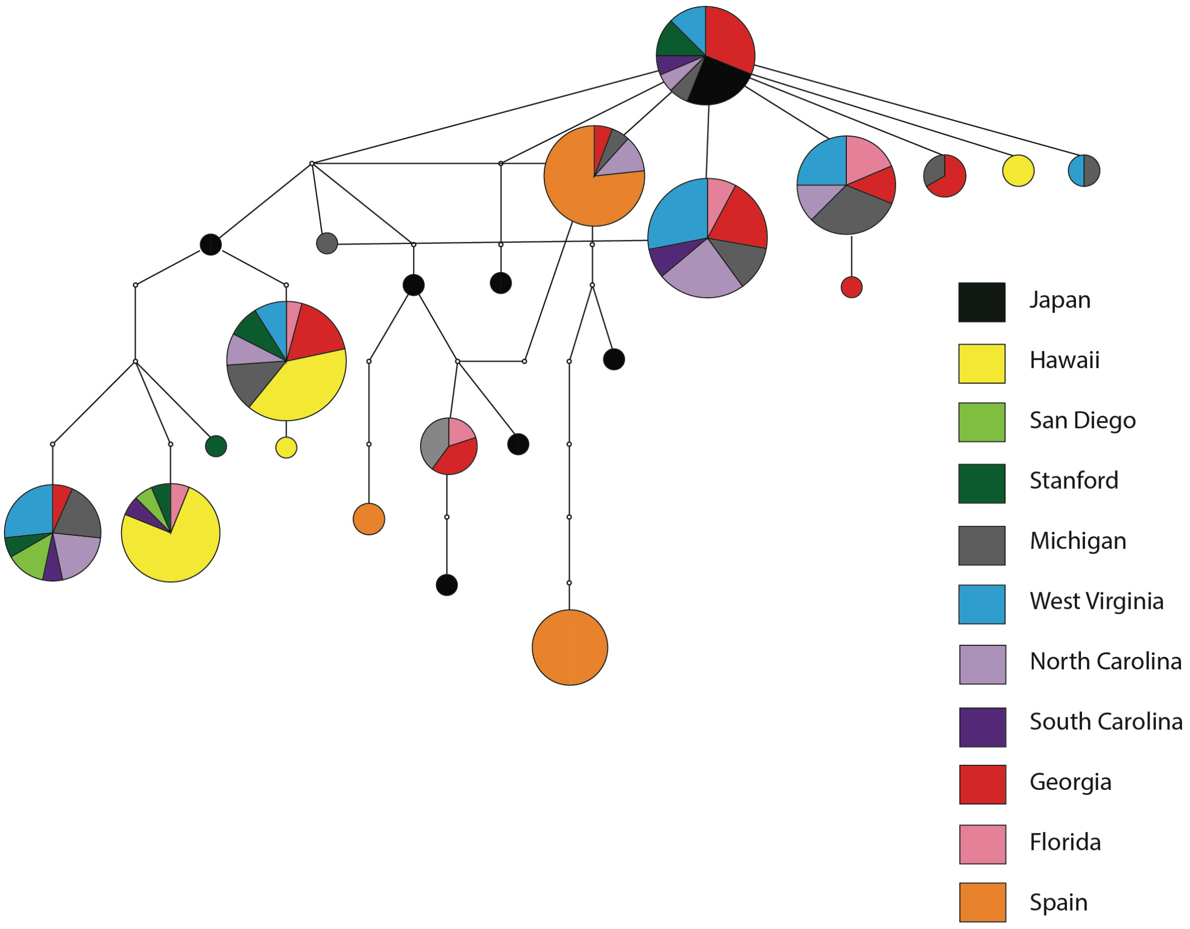

Supplementary Figure 5: Haplotype network for locus 30437

Supplementary Figure 6
a)

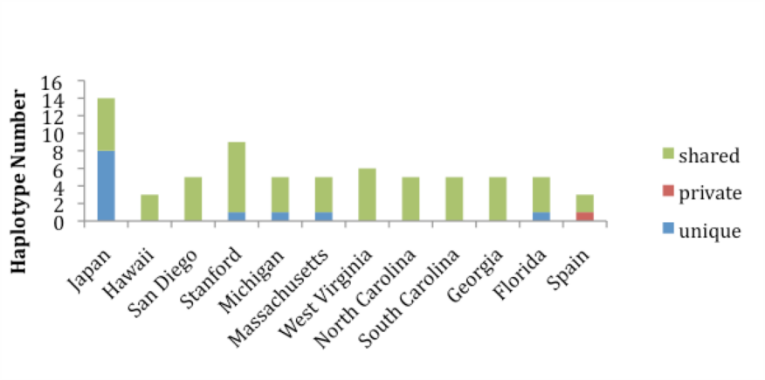

b)

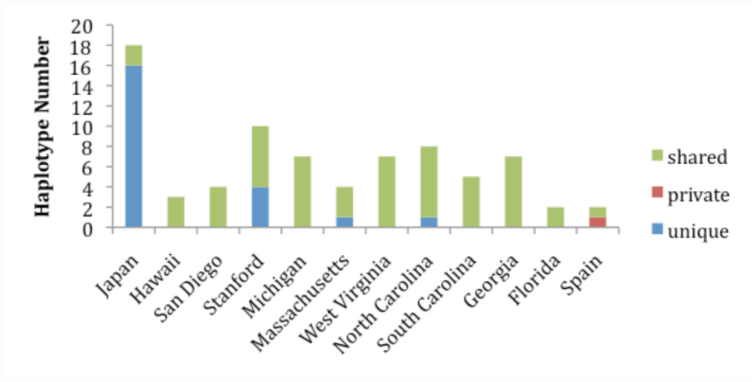

c)

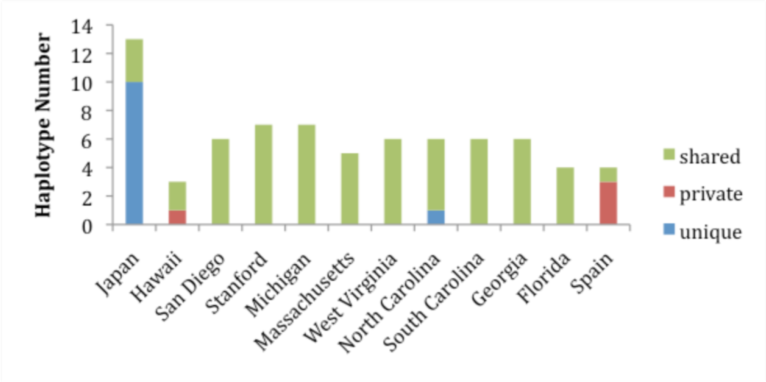

d)

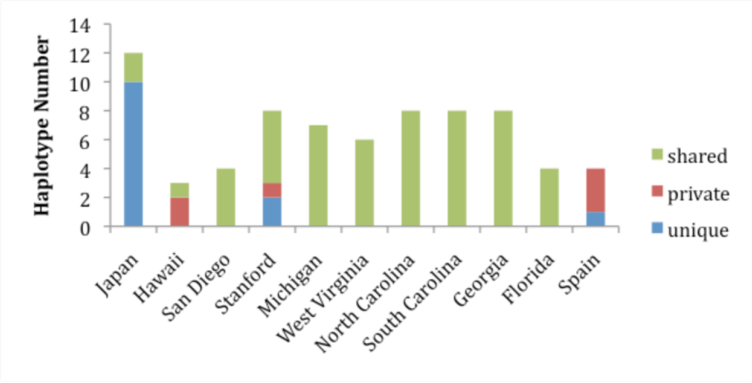

e)

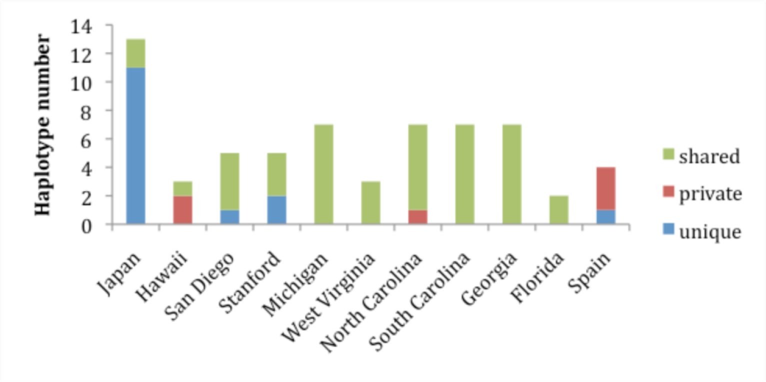

f)

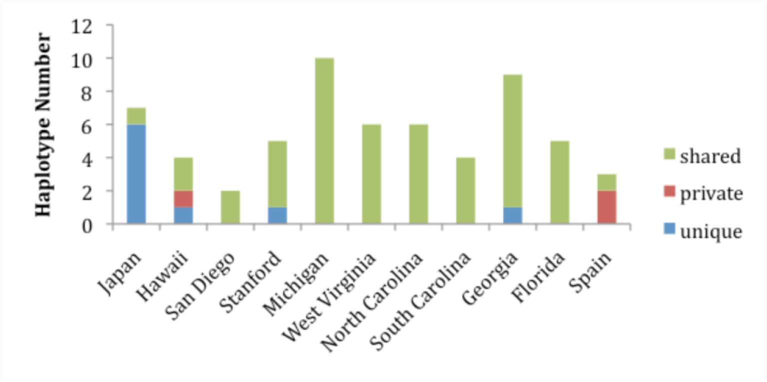

Supplementary Figure 6: Total number of shared (green), private (red) and unique (blue) haplotypes in each population for locus a) 29789, b) 29997, c) 26206, d) 17561, e) 1083, and f) 30437.

Supplementary Figure 7

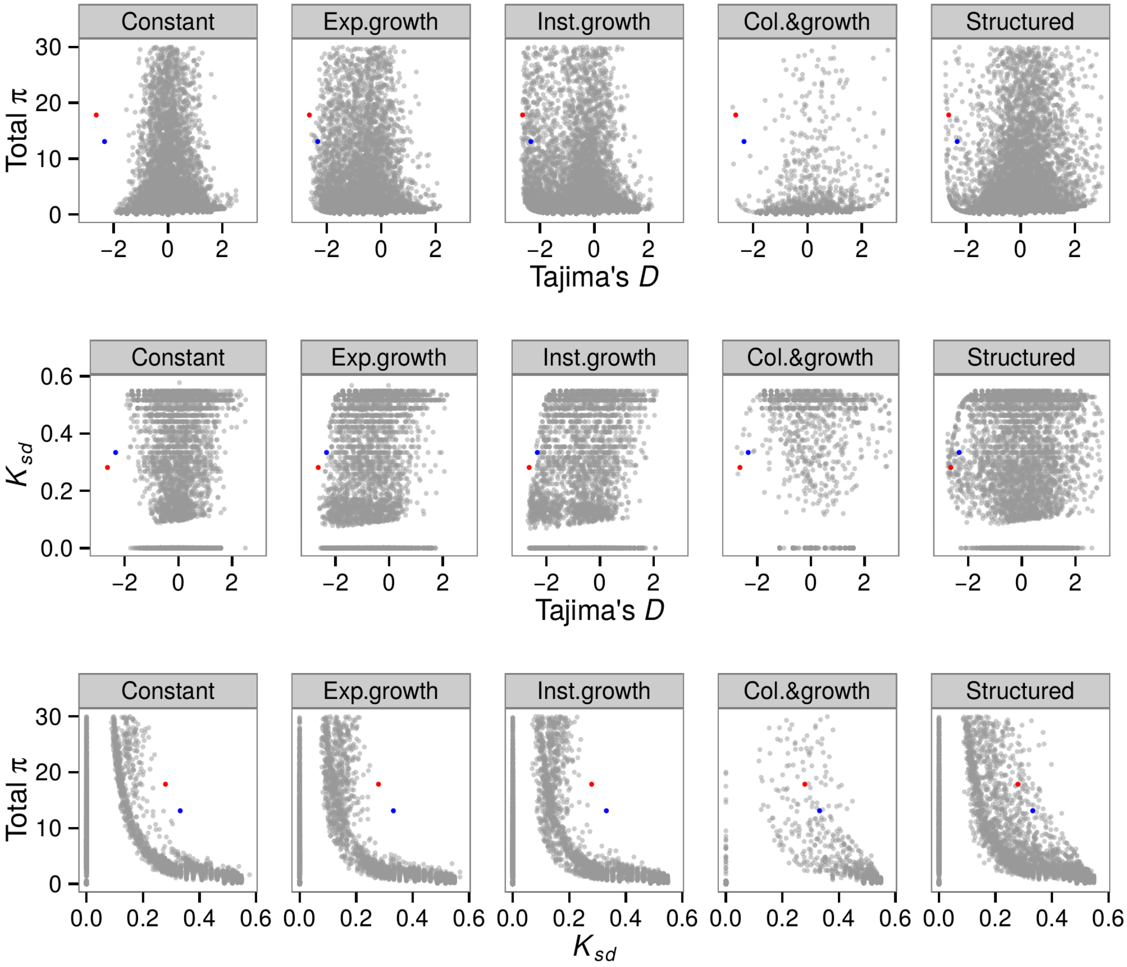

Supplementary Figure 7. The predicted π, Tajima's D and K_{sd} values under different parameter combinations from the five tested models for the Japanese population. The observed data for all sites and non-coding sites only are shown in red and blue respectively.

Supplementary Figure 8

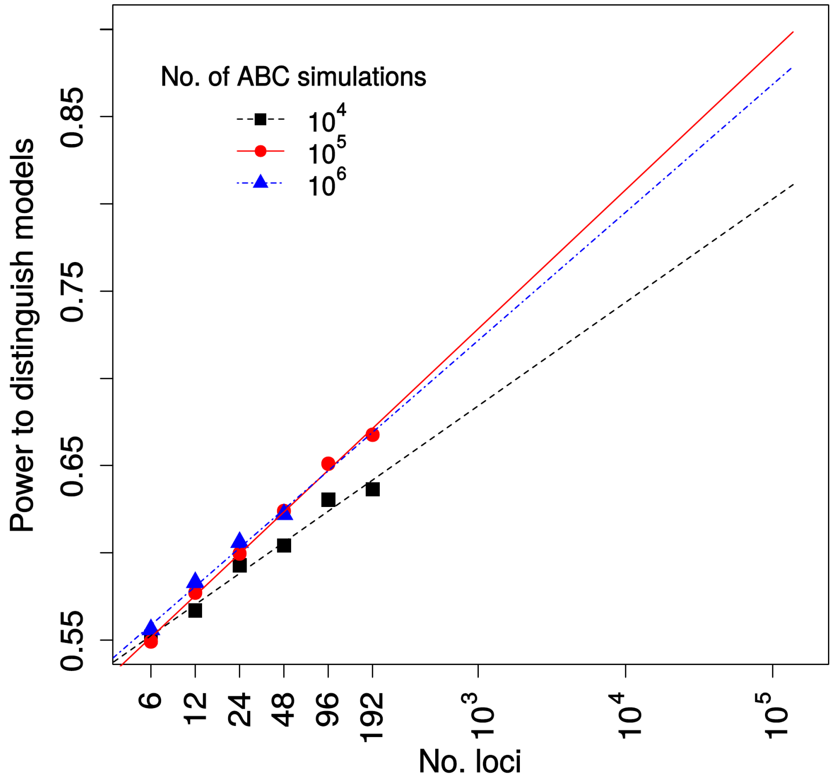

Supplementary Figure 8. The relationship between number of loci and statistical power to distinguish the 3 colonization models for *D. suzukii* using 10^4, 10^5 and 10^6 ABC simulations. Model choice was performed on 10^3 retained simulations in all cases. Points denote results of simulations and line is the fitted linear regression. The x-axis (number of loci) is $\log_{10}$-scaled.

Supplementary Figure 9

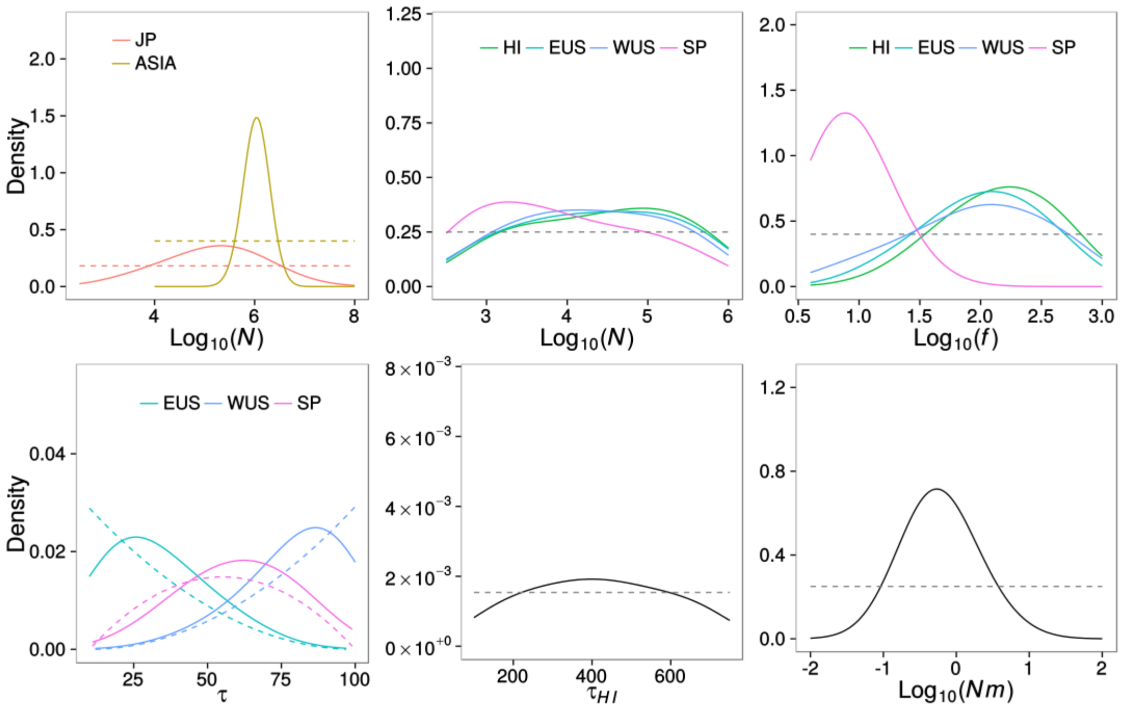

Supplementary Figure 9. The posterior probability of the parameters of the 3 colonization models for *D. suzukii* inferred using noncoding sites only and weighted by the posterior probability of each model.

Supplementary Figure 10

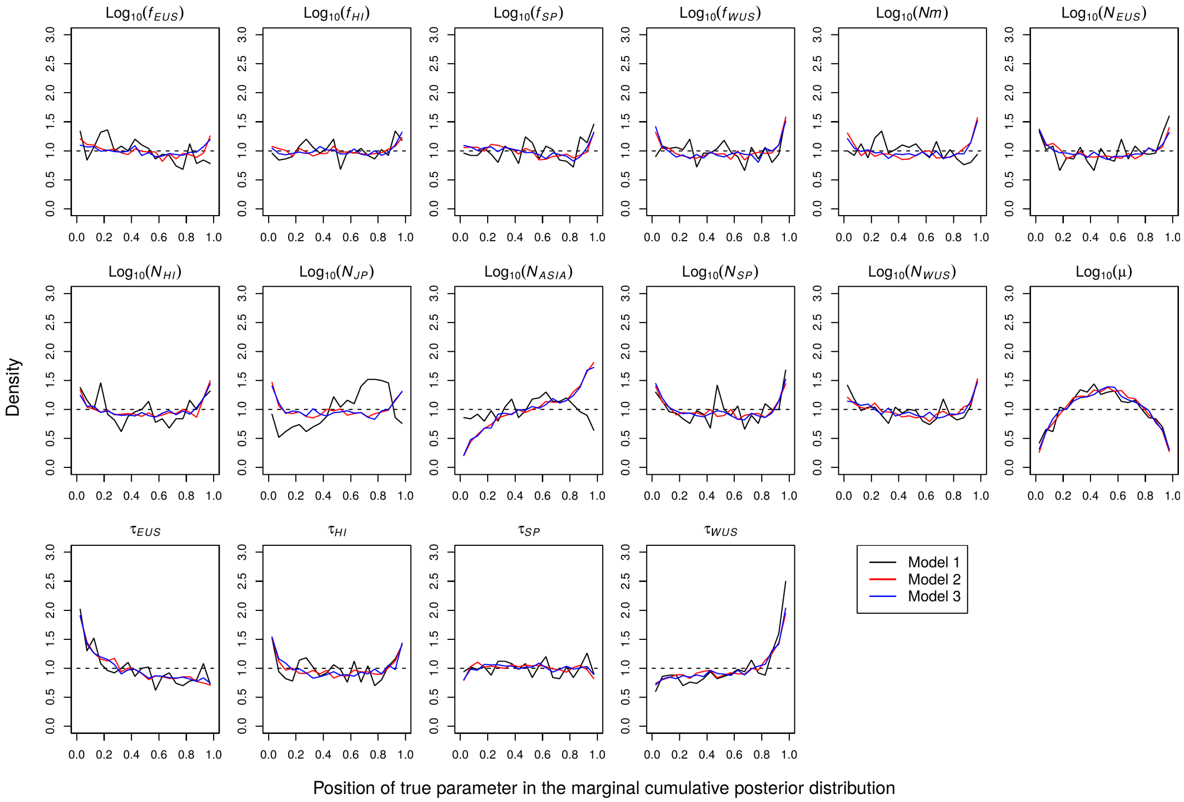

Supplementary Figure 10. The distribution of the position of the true parameter in the marginal cumulative posterior distribution for each model.

Supplementary Figure 11

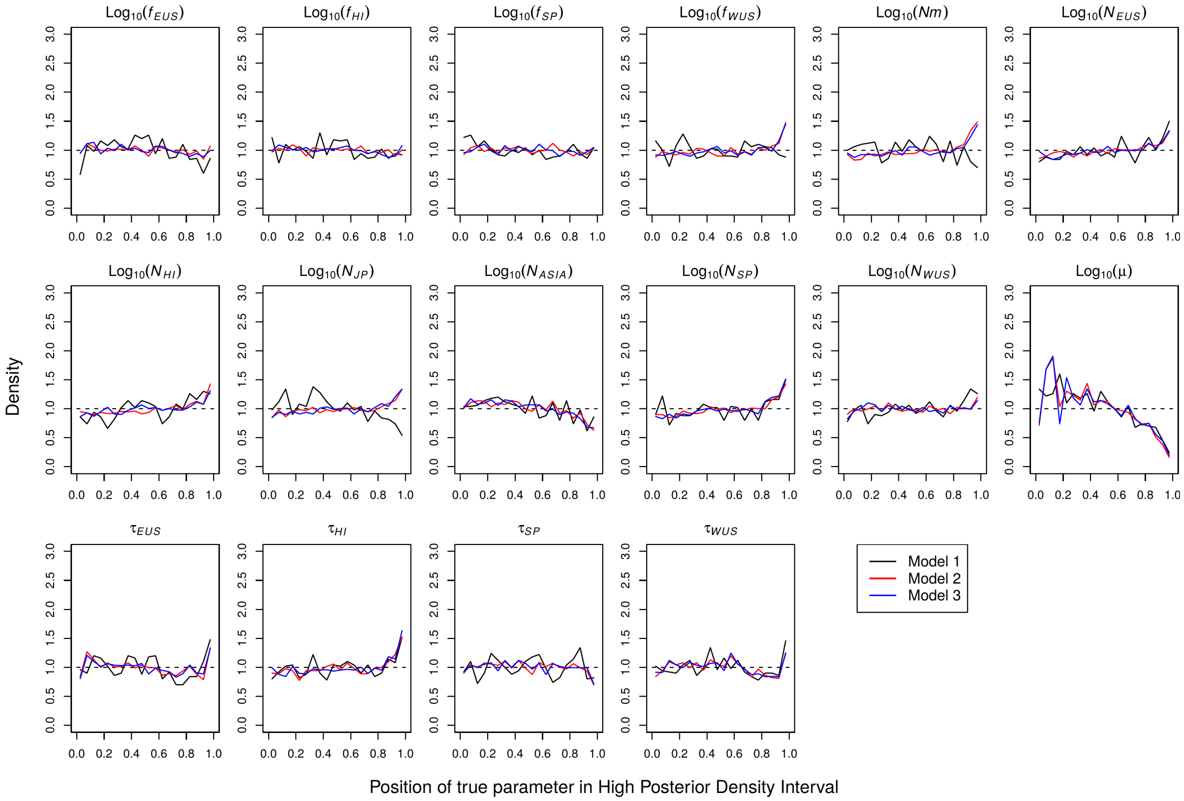

Supplementary Figure 11. The distribution of the smallest high posterior density Interval (HPDI) containing the true parameter value for each model.

Table S1: Primer sequences and physical locations in *D. melanogaster*

Primer	Physical Position (Mbp)	Sequence
29789L	5.6	AACCTGGACATCTACGATCC
29789R		CAATCAGTAGAACGCAGACC
29997L	8.0	GACATGAAGACGCACTTCTC
29997R		TAGAACATACCAGCCGTGTC
26206L	9.0	TGCAGATATTCTCCAAGTGC
26206R		GGAAAGCAAGTGACCTTCTC
17561L	10.7	TCTACAGGCGCTACAAAAAG
17561R		TCAGATAACCCACATCCTTG
1083L	11.9	GTACGGACGTACCCTTATCC
1083R		CTCTGGAGCTTCGTGACTC
30437L	12.9	CGATAACGAAGGGAATTTG
30437R		ACATGCAAGAATGTTTAGCC

Table S2. Priors for the five models of the Japanese population.

Model(s)	Parameter	Prior
Constant, Exponential growth, Instantaneous growth	$\text{Log}_{10}(N_0)$	U[4,8]
Exponential growth, Instantaneous growth	$\text{Log}_{10}(N_t/N_0)$	U[0,4]
	$\text{Log}_{10}(\tau/N_t)$	U[-2,2]
Colonization & exponential growth	$\text{Log}_{10}(N_{ASIA})$	U[4,8]
	$\text{Log}_{10}(f_{JP})$	U[0.6,3]
	$\text{Log}_{10}(N_{JP})$	U[2,6]
	τ	$U[10^3,10^4]$
Structured	$\text{Log}_{10}(N_{ASIA})$	U[4,8]
	$\text{Log}_{10}(N_{JP})$	U[2,6]
	$\text{Log}_{10}(Nm)$	U[-2,2]
All	$\mu \times 10^9$	N(3.46,0.28)

N_0: ancestral JP size, N_t/N_0: size of expansion, τ/N_t: time of expansion relative to N_t, N_{ASIA}: size of source population, f_{JP}: no. of founders for JP, N_{JP}: current size of JP, τ: time of colonization in generations backwards in time, Nm: migration rate, μ: mutation rate

Table S3. Reported are the posterior probabilities for models of the Japanese population of *D. suzukii*. The model with the highest probability within a set is shown in bold. The *P*-value for the observed data, the Tukey depth and the *P*-value for a Tukey test are reported.

Model	Posterior probability	Observed *P*-value	Tukey depth	Tukey *P*-value
Constant	2.26×10^{-16}	0.002	0.004	0.776
Exponential growth	3.85×10^{-8}	0.008	0	0
Instantaneous growth	4.29×10^{-10}	0.536	0.418	0.829
Colonization and exp. growth	1.10×10^{-4}	0.018	0.028	0.860
**Structured population**	**0.999**	**0.896**	**0.225**	**0.681**

Table S4. Expected number of mutations among 1000 loci as a function of the sample size for different parameter combinations of the number of founders *f* and the time since colonization τ (see Figure 7 for the actual values). Fold change when compared to the case of 25 samples is given in parenthesis.

		Scenario											
Sample size		A1		A2		B1		B2		C1		C2	
25	(1.0)	3.9	(1.0)	15.4	(1.0)	1.5	(1.0)	7.1	(1.0)	0.4	(1.0)	1.8	(1.0)
50	(2.0)	6.5	(1.7)	22.9	(1.5)	2.7	(1.8)	11.7	(1.6)	0.7	(1.8)	3.5	(1.9)
100	(4.0)	10.3	(2.6)	32.9	(2.1)	4.6	(3.1)	18.3	(2.6)	1.3	(3.3)	6.2	(3.4)
200	(8.0)	15.9	(4.1)	45.3	(2.9)	7.6	(5.1)	27.2	(3.8)	2.4	(6.0)	10.6	(5.9)
